# An Operating-Consistency and Evidence-Refinement Framework for Sensor-Data-Driven Photovoltaic Panel Risk Assessment and Maintenance Prioritization

**DOI:** 10.3390/s26144487

**Published:** 2026-07-15

**Authors:** Zheng Tang, Chenhao Sun, Xuejun Ren, Xiaoshuang Zhang, Jie Zhu, Haochen Li

**Affiliations:** State Key Laboratory of Disaster Prevention and Reduction for Power Grid, Changsha University of Science and Technology, Changsha 410114, China; tangzheng042311@163.com (Z.T.); renxuejun31@gmail.com (X.R.); 15118177122@163.com (X.Z.); m17774684526@163.com (J.Z.); osxarlihao@gmail.com (H.L.)

**Keywords:** photovoltaic monitoring, monitoring data analytics, risk assessment, operating consistency, evidence refinement, maintenance prioritization

## Abstract

Photovoltaic plants generate large amounts of electrical, thermal, environmental, and equipment-status monitoring data during long-term operation. These data provide an important basis for risk assessment and maintenance decision making, but their practical use remains challenging because photovoltaic output is strongly affected by irradiance, temperature, and environmental fluctuation. Conventional threshold-based schemes are sensitive to operating conditions, while common data-driven classifiers mainly focus on label prediction and provide limited support for operating-consistency interpretation, calibrated risk assessment, and maintenance-priority ranking. To address these issues, an Operating Consistency and Evidence Refinement Framework, named OCERF, is proposed for photovoltaic panel risk assessment. First, photovoltaic generation records, weather-sensor observations, thermal measurements, and state-level monitoring information are transformed into a unified risk-evidence matrix through missing-data handling, normalization, risk-direction alignment, and discrete-state risk encoding. Unlike direct feature concatenation, an operating-consistency residual autoencoding model is used to learn the normal relationship among electrical output, environmental response, thermal state, and efficiency-related variables, so that abnormal deviations can be identified through reconstruction residuals. Since continuous deviations may also be caused by normal environmental fluctuation, candidate-constrained discrete-state refinement is further introduced to strengthen the credibility of continuous abnormal samples. Finally, instead of using a simple weighted sum, a CD-MABAC composite ranking model integrates continuous risk, discrete enhancement, and continuous-discrete consistency, and logistic calibration is used to obtain calibrated reference-risk probabilities and risk grades. Evaluations on photovoltaic generation and weather-sensor data show improved high-risk identification, probability calibration, and ranking stability. The results indicate that OCERF can support intelligent photovoltaic monitoring, inspection prioritization, and maintenance management under limited operation and maintenance resources.

## 1. Introduction

### 1.1. Research Background

With the continuous expansion of renewable energy installations, photovoltaic power generation has become an important component of modern power systems. Photovoltaic plants have been widely deployed in centralized power stations, distributed energy systems, microgrids, and smart distribution networks because of their clean energy characteristics, short construction period, and relatively low operation cost [1,2,3]. Meanwhile, the operation and maintenance of photovoltaic plants are increasingly supported by monitoring systems that collect electrical, thermal, environmental, and equipment-status data. Typical monitoring information includes DC voltage, DC current, output power, solar irradiance, ambient temperature, module temperature, humidity, wind speed, inverter status, alarm records, and maintenance records. These multisource monitoring data provide a basis for operating-state perception, abnormality identification, and maintenance decision making.

However, photovoltaic modules are exposed to complex outdoor environments for long periods. Their operating states can be affected by solar irradiance fluctuation, ambient temperature variation, dust accumulation, partial shading, hot spot formation, module aging, string mismatch, and inverter-side abnormality [4,5,6,7]. These factors may reduce generation efficiency and may also lead to abnormal output power, temperature rise, local degradation, or potential safety risks. Therefore, accurate risk assessment and early warning of photovoltaic operating states are of great importance for improving generation efficiency, reducing maintenance cost, and ensuring safe operation of photovoltaic plants [8,9,10].

A key difficulty in photovoltaic risk assessment is that abnormal states cannot be reliably identified from a single monitoring indicator. For example, low output power under weak irradiance does not necessarily indicate module degradation, whereas similar low output under sufficient irradiance may correspond to module aging, shading, dust accumulation, or string abnormality. Similarly, an increase in module temperature may be jointly influenced by ambient temperature, irradiance intensity, heat dissipation conditions, and internal module degradation. Therefore, photovoltaic risk assessment should not be based only on the magnitude of a single indicator. The operating consistency among electrical output, environmental response, thermal condition, and efficiency variation should also be considered [11,12,13,14,15].

Conventional photovoltaic operation and maintenance mainly depends on periodic manual inspection, empirical threshold judgment, and performance ratio analysis. These methods are simple to implement and have relatively clear engineering meanings. However, their assessment results are easily affected by fixed thresholds and changing operating conditions [11,12]. In practical photovoltaic plants, the same output-power value or temperature value may have different risk meanings under different irradiance, weather, and operating conditions. Therefore, a more reliable risk assessment model should be able to describe whether the relationships among multiple monitoring variables are consistent with normal operating patterns.

In practical photovoltaic plants, maintenance resources are usually limited, and it is difficult for operators to inspect all modules or operating samples with the same priority. Therefore, it is not sufficient to simply classify samples as normal or abnormal. A more useful risk assessment model should provide interpretable reference risk probabilities and maintenance priority rankings, so that samples with higher potential risk can be inspected and treated first [16,17]. From this perspective, photovoltaic risk assessment is not only a classification problem, but also a comprehensive decision problem involving heterogeneous monitoring-data representation, operating-consistency modeling, risk evidence refinement, probability calibration, and interpretable priority ranking.

### 1.2. Related Work and Existing Limitations

Existing studies on photovoltaic fault diagnosis and risk assessment can generally be divided into three categories.

The first category includes physical rule-based and empirical threshold-based methods. In these approaches, the operating state of a photovoltaic system is evaluated using irradiance, output power, module temperature, conversion efficiency, performance ratio, and power deviation [11,12,13]. Abnormal conditions are identified when output power significantly deviates from expected values or when thermal and efficiency indicators exceed predefined thresholds. These methods are computationally efficient and provide strong engineering interpretability, making them suitable for rapid screening of abnormal generation, thermal anomalies, and efficiency degradation. However, threshold rules are typically determined empirically and exhibit limited adaptability across varying seasons, weather conditions, irradiance levels, and plant configurations [14,15]. In addition, most threshold-based methods evaluate variables independently and fail to capture operating-consistency deviations among coupled photovoltaic variables.

The second category includes data-driven photovoltaic fault diagnosis and anomaly detection methods. In recent years, machine learning and deep learning models such as support vector machines, random forests, neural networks, convolutional neural networks, recurrent neural networks, long short-term memory networks, and autoencoders have been widely applied to photovoltaic condition monitoring [18,19,20,21,22,23,24,25]. These methods extract nonlinear representations from electrical measurements, environmental data, and image-based inspection data, achieving promising performance in fault identification and anomaly detection tasks [25,26,27]. For instance, infrared thermography and electroluminescence imaging have been used for hotspot and defect detection, while time-series models detect output and efficiency anomalies through learned representations [20,24]. However, most existing methods are designed primarily for classification accuracy or anomaly detection. Their outputs are often difficult to interpret as calibrated risk probabilities or maintenance priority rankings. Moreover, limited availability of labeled fault data further restricts supervised learning performance, leading to issues such as label dependence and weak generalization across operating conditions [28,29].

The third category involves multi-criteria decision-making (MCDM) and risk ranking methods. MCDM techniques transform multiple indicators into interpretable composite scores or rankings and have been widely applied in energy system evaluation and maintenance decision support [30,31,32,33]. Common approaches include AHP, TOPSIS, VIKOR, MABAC, and CRITIC-based weighting strategies [34,35,36,37,38]. These methods are effective in producing interpretable ranking results under limited maintenance resources. However, most existing approaches directly aggregate predefined indicators without explicitly modeling operating-consistency relationships among continuous photovoltaic variables. In addition, discrete-state evidence such as temperature levels, power deviation states, alarm records, or maintenance logs is typically used in a static manner, without considering its enrichment behavior within high-risk candidate samples. As a result, such methods may struggle to distinguish transient environmental fluctuations from true degradation-induced risk.

Recently, explainable artificial intelligence (XAI) and transformer-based architectures have attracted increasing attention in photovoltaic monitoring and fault diagnosis. These models aim to capture long-range temporal dependencies and nonlinear dynamics in photovoltaic time-series data [39,40,41]. In addition, XAI techniques such as SHAP-based feature attribution and global explanation methods improve model interpretability by quantifying feature contributions to predictions [42,43,44]. More recently, self-supervised learning and foundation-model-inspired approaches have been explored for time-series modeling, where representation learning from large-scale unlabeled data enhances robustness under limited supervision and distribution shifts [45,46]. However, most existing approaches still focus on classification or anomaly detection tasks, where outputs are limited to binary or categorical decisions. They rarely integrate continuous operating consistency modeling, discrete-state evidence refinement, and interpretable risk ranking within a unified decision framework.

In addition to fault identification and ranking ability, probability reliability is also important in photovoltaic risk assessment. For maintenance decision making, model outputs should not only provide relative risk scores, but should also be consistent with observed high-risk frequencies as much as possible. The ROC curve, PR curve, Brier Score, expected calibration error, and calibration curve have been widely used to evaluate model discrimination ability and probability calibration quality [47,48,49,50,51]. However, in many photovoltaic risk assessment studies, model outputs are mainly used for classification, while probability calibration and Top k maintenance priority evaluation have received relatively limited attention. As a result, it is difficult for such models to directly answer the practical operation and maintenance question of which samples should be inspected first.

In summary, several limitations remain in existing studies.

- First, photovoltaic operating data usually contain both continuous variables and discrete state variables, but a unified risk representation for these heterogeneous features is still insufficient.
- Second, photovoltaic output power and module temperature are significantly affected by environmental factors. If only the magnitude of a single indicator is considered, misjudgment may occur. Continuous risk should be characterized from the perspective of operating consistency deviation.
- Third, discrete state evidence is often simply encoded or directly used as model input, while its enrichment among continuous high risk candidate samples is rarely considered.
- Fourth, many existing methods focus more on classification performance than on probability calibration and maintenance priority ranking under limited inspection resources.

Therefore, a photovoltaic risk assessment framework is still needed to integrate continuous and discrete risk evidence, characterize operating consistency deviation, refine state level risk information, and provide interpretable risk ranking results.

### 1.3. Statement and Contributions

OCERF is designed as a unified risk-evidence modeling framework rather than a combination of anomaly detection, feature fusion, and ranking modules. Unlike traditional pipelines that sequentially execute independent algorithms, OCERF defines a single modeling process in which heterogeneous sensor observations are consistently mapped into structured risk evidence for decision-making.

To address the challenges of sensor-data-driven photovoltaic monitoring, OCERF integrates continuous risk screening, discrete-state refinement, and risk ranking within a unified transformation process. These components are not independent tasks, but successive stages of abstraction within a shared risk-evidence space, where raw measurements are progressively encoded into consistency-aware representations and ultimately transformed into calibrated risk priorities.

Overall, OCERF emphasizes a risk-evidence transformation paradigm that jointly models feature representation and decision-level ranking in a single coherent framework rather than a sequential execution of separate modules.

The methodological novelty of this study does not lie in proposing a new standalone classifier, but in constructing a risk-evidence serial refinement and decision-ranking framework for photovoltaic operation and maintenance. This serial design is adopted because continuous deviations in photovoltaic operation may be caused by normal environmental fluctuations. For example, low output power may be normal under weak irradiance, but it becomes more credible as a risk signal when accompanied by high temperature level, large power-deviation state, output-abnormality state, or other available abnormal records. Therefore, discrete-state evidence is used as a subsequent refinement rather than as a simple parallel input. The final ranking score is further calibrated into an interpretable reference-risk probability and converted into risk grades and maintenance priorities, which supports high-risk sample identification under limited inspection resources.

This study focuses on sensor-data-driven operational risk estimation rather than fault diagnosis with ground-truth labels, where risk labels are constructed as operational proxies from observed operating deviations.

The main contributions of this study are summarized as follows.

- A heterogeneous photovoltaic risk matrix is constructed to unify continuous operating variables and discrete state variables into a risk evidence space. Through missing data handling, normalization, risk direction alignment, and discrete state risk encoding, multi source photovoltaic features are represented in a unified form, providing a consistent input for subsequent continuous risk screening and discrete state refinement.
- A continuous risk screening and discrete state evidence refinement mechanism is proposed. An operating consistency residual autoencoding model is used to learn the normal operating relationship among continuous photovoltaic variables, and reconstruction residuals are used to characterize the deviation of each sample from the normal operating pattern. On this basis, a candidate constrained discrete state enrichment strategy is designed to quantify the risk enhancement effect of discrete states on continuous high risk candidate samples.
- A CD MABAC based composite risk ranking model is developed. Continuous risk, discrete state enhancement, and continuous discrete consistency are treated as three risk criteria, and the composite risk ranking score is calculated according to the deviation from the border approximation area. Logistic calibration and risk grade classification are further introduced to provide calibrated risk probabilities, graded warning results, and maintenance priority rankings.
- A systematic empirical evaluation is conducted using photovoltaic generation records and weather sensor data. ROC, PR AUC, Brier Score, ECE, confusion matrix, Precision at k, Hit at k, Lift at k, ablation study, and sensitivity analysis are used to evaluate the discrimination ability, probability reliability, ranking performance, and robustness of the proposed framework. The experimental results show that OCERF can identify and rank high risk photovoltaic samples more effectively under limited inspection resources.

## 2. Methods

### 2.1. Construction of the Heterogeneous Risk Matrix for Photovoltaic Panels

The proposed OCERF framework is not a conventional feature engineering or classification pipeline. Instead, it defines a structured risk-evidence modeling process that progressively transforms heterogeneous sensor data into decision-ready risk representations.

To support graded identification of latent defects and interpretable risk prioritization for photovoltaic panels, a heterogeneous risk matrix is first constructed for photovoltaic operating scenarios. The operating condition of a photovoltaic panel is generally affected by multiple factors, including electrical output characteristics, external environmental conditions, module surface states, equipment operating states, and fault or alarm events. If only a single type of feature is used, potential degradation, low-generation abnormality, thermal anomaly, or surface shading risk cannot be sufficiently characterized. Therefore, before risk modeling, continuous monitoring variables and discrete state variables are organized into a sample-level hybrid feature matrix and further transformed into a risk-evidence matrix with unified risk direction. This matrix provides a consistent input basis for subsequent continuous-feature risk screening, discrete-state refinement, and composite risk scoring.

The representative heterogeneous features considered for photovoltaic-panel risk assessment are listed in Table 1.

As shown in Table 1, the feature system used for photovoltaic-panel risk assessment contains both continuously varying electrical and environmental variables and symbolically represented state variables. The continuous features describe the output capability and operating deviation of photovoltaic panels under different irradiance and temperature conditions, whereas the discrete features provide structural information related to surface abnormality, equipment-state change, alarm events, and maintenance behavior. These two types of features jointly form the input basis for subsequent risk screening and risk ranking.

#### 2.1.1. Missing-Data Handling

In practical photovoltaic data acquisition, missing entries may be caused by sensor abnormality, communication interruption, asynchronous sampling, extreme weather conditions, or incomplete maintenance records. To ensure data quality, a combined strategy of proportional-threshold deletion and type-specific imputation is adopted. Specifically, if the missing ratio of a feature exceeds a preset threshold δ, the feature is removed from the candidate feature set. In this study, δ is set to 5%. For the remaining incomplete entries, imputation is performed according to the feature type: mean imputation is applied to continuous variables, and mode imputation is applied to discrete variables. In this way, sample completeness can be maintained while the influence of missing values on the subsequent construction of the risk matrix is reduced.

In addition, the daytime and nighttime periodicity of photovoltaic generation is considered. Low power output under low-irradiance or zero-irradiance conditions does not necessarily indicate a module fault. Therefore, invalid generation periods are identified before risk-sample construction. In general, samples collected when irradiance is close to zero or when the photovoltaic system is under natural non-generating conditions are excluded from low-generation risk judgment, so that nighttime shutdown or naturally low output under extremely weak irradiance is not incorrectly treated as a fault-related risk.

To improve robustness under real-world photovoltaic operating conditions, preprocessing steps are incorporated to mitigate the effects of missing data and sensor noise within the data preparation stage. Missing values are handled through consistency-preserving interpolation, while measurement noise is reduced using smoothing and aggregation operations during feature construction. Based on the processed data, subsequent risk-related feature extraction is performed.

#### 2.1.2. Construction of the Hybrid Feature Matrix

In this study, the label ci represents an operational risk proxy rather than a ground-truth fault annotation. Due to the absence of real fault and maintenance records in the dataset, risk labels are constructed based on observed deviations in photovoltaic operating behavior. Specifically, these proxy labels are derived from physically meaningful operating deviations, including power output deviation, irradiance consistency, and temperature-efficiency mismatch patterns.

After missing data handling, all sample records are organized into a hybrid feature matrix. Let

(1) $$\begin{array}{c} L=\left\{c_{1},c_{2},\ldots ,c_{m}\right\} \end{array}$$

denote the label set corresponding to m photovoltaic-panel samples, where $c_{i}$ represents an operational risk label derived from constructed proxy rules rather than a fault-state label. of the i-th sample. Let

(2) $$\begin{array}{c} A=\left[a_{ij}\right],i=1,2,\ldots ,m;j=1,2,\ldots ,n \end{array}$$

denote the input feature matrix, where each row corresponds to one sample and each column corresponds to one feature variable. Let

(3) $$\begin{array}{c} P=\left\{p_{1},p_{2},\ldots ,p_{m}\right\} \end{array}$$

denote the target variable set, where $p_{i}$ represents the risk assessment score or graded risk level of the i-th sample. Based on these definitions, the hybrid feature matrix is expressed as

(4) $$\begin{array}{c} M=\left[\begin{array}{cccccc} c_{1} & a_{11} & a_{12} & \ldots & a_{1n} & p_{1}\\ c_{2} & a_{21} & a_{22} & \ldots & a_{2n} & p_{2}\\ \vdots & \vdots & \vdots & \ddots & \vdots & \vdots \\ c_{m} & a_{m1} & a_{m2} & \ldots & a_{mn} & p_{m} \end{array}\right] \end{array}$$

In this matrix, the first column represents the sample label vector L, the middle part corresponds to the input feature matrix A, and the last column represents the target variable vector P. The feature matrix A contains both continuous and discrete photovoltaic-panel attributes. Specifically, the continuous features include electrical and environmental variables such as DC voltage, DC current, output power, power deviation, solar irradiance, ambient temperature, and module temperature. The discrete features include surface condition, shading state, dust level, inverter status, alarm type, fault type, and maintenance flag.

For subsequent modeling, the input feature matrix A is further decomposed into continuous and discrete submatrices:

(5) $$\begin{array}{c} X=\left[X^{\left(c\right)},X^{\left(d\right)}\right] \end{array}$$

where

(6) $$\begin{array}{c} X^{\left(c\right)}\in R^{m\times p} \end{array}$$

denotes the continuous feature submatrix, and

(7) $$\begin{array}{c} X^{\left(d\right)}\in D^{m\times q} \end{array}$$

denotes the discrete feature submatrix, with p+q=n. Each row of X corresponds to one photovoltaic-panel sample, and each column corresponds to one feature variable. The role of M is to describe the complete sample organization including labels, features, and target variables, while X is the actual input feature matrix extracted from M for subsequent risk modeling. Therefore, X does not replace M; instead, it is the feature component of M.

Because continuous and discrete features differ substantially in numerical range, statistical distribution, and semantic interpretation, directly feeding X into a model may lead to representation bias. High-magnitude continuous variables may dominate the optimization process, while discrete state variables may fail to express their true risk contribution. Therefore, before sequential risk modeling, all features are transformed into a unified risk-oriented representation space.

#### 2.1.3. Normalization and Unified Risk-Oriented Encoding

For continuous features, min–max normalization is first applied:

(8) $$\begin{array}{c} {\tilde{x}}_{ij}^{\left(c\right)}=\frac{x_{ij}^{\left(c\right)}-min(x_{j}^{\left(c\right)})}{max(x_{j}^{\left(c\right)})-min(x_{j}^{\left(c\right)})+\varepsilon } \end{array}$$

where $x_{ij}^{\left(c\right)}$ denotes the value of the j-th continuous feature in the i-th sample, and ε is a small constant used to avoid division by zero. Since different continuous variables may have different risk directions, risk-direction alignment is further performed:

(9) $$\begin{array}{c} r_{ij}^{\left(c\right)}=\left\{\begin{array}{cc} {\tilde{x}}_{ij}^{\left(c\right)}, & \mathrm{if}\;\mathrm{feature}\;j\;\mathrm{is}\;\mathrm{positively}\;\mathrm{correlated}\;\mathrm{with}\;\mathrm{risk},\\ 1-{\tilde{x}}_{ij}^{\left(c\right)}, & \mathrm{if}\;\mathrm{feature}\;j\;\mathrm{is}\;\mathrm{negatively}\;\mathrm{correlated}\;\mathrm{with}\;\mathrm{risk}. \end{array}\right. \end{array}$$

For example, higher module temperature, larger power deviation, and abnormal current fluctuation usually indicate higher risk, whereas higher conversion efficiency or stable output performance usually indicates lower risk. After risk-direction alignment, all continuous variables follow the same convention that larger values correspond to higher potential risk.

For discrete features, simple one-hot encoding is not adopted because it only preserves category differences but does not directly reflect risk semantics. Instead, each categorical state is mapped to a risk-related numerical value according to its empirical association with high-risk samples. For the k-th discrete feature and one of its states c, the empirical risk score is defined as

(10) $$\begin{array}{c} u_{k}\left(c\right)=\frac{n_{k}^{+}(c)+\lambda \pi }{n_{k}(c)+\lambda } \end{array}$$

where $n_{k}(c)$ denotes the number of samples taking state c, $n_{k}^{+}(c)$ denotes the number of high-risk samples among them, π denotes the overall high-risk sample ratio in the training set, and λ is a smoothing coefficient. To ensure comparability across different discrete features, the encoded values are further normalized as

(11) $$\begin{array}{c} r_{ik}^{\left(d\right)}=\frac{u_{k}(c_{ik})-min(u_{k})}{max(u_{k})-min(u_{k})+\varepsilon }, \end{array}$$

where $c_{ik}$ denotes the categorical state of the k-th discrete feature in the i-th sample.

Based on the above transformations, the final unified risk-evidence matrix is obtained as

(12) $$\begin{array}{c} R=\left[R^{\left(c\right)},R^{\left(d\right)}\right], \end{array}$$

where $R^{\left(c\right)}$ denotes the continuous risk-evidence submatrix and $R^{\left(d\right)}$ denotes the discrete risk-evidence submatrix. All columns in R follow the same risk-oriented convention, namely, larger values indicate higher potential risk. It should be noted that this unified matrix is not the final decision result. Instead, it provides a consistent input basis for the subsequent sequential framework: continuous risk evidence is first used to construct the base risk state, and discrete risk evidence is then introduced to refine the base representation through surface-state, fault-event, and maintenance-pattern information.

### 2.2. Continuous Risk Screening Based on Operating Consistency Residual Autoencoding

After the heterogeneous risk matrix has been constructed, potential high risk samples are first screened from continuous operating features. The continuous features of photovoltaic panels mainly include voltage, current, power, energy generation, solar irradiance, ambient temperature, module temperature, power deviation, and conversion efficiency. These variables directly reflect the output capability, thermal condition, and efficiency variation of photovoltaic panels under different external conditions, and are therefore important for identifying low generation abnormality, temperature rise abnormality, and efficiency degradation.

The continuous features of photovoltaic panels are not independent of each other. Instead, they are jointly constrained by the operating mechanism of photovoltaic generation. Under normal operating conditions, a relatively stable relationship should exist among irradiance, module temperature, output power, and conversion efficiency. For example, when irradiance is sufficient and module temperature remains within a reasonable range, the output power is expected to remain within a corresponding operating interval. If sufficient irradiance is accompanied by significantly reduced output power, or if increased module temperature is accompanied by decreased conversion efficiency, the sample may have deviated from the normal operating state. Based on this characteristic, a residual autoencoding model is adopted to learn the normal operating consistency among continuous features. The reconstruction residual is then used to characterize the degree to which a sample deviates from the normal operating manifold, so that preliminary risk screening can be performed at the continuous feature level.

#### 2.2.1. Continuous Feature Input and Operating Consistency Assumption

Let the normalized continuous feature matrix before risk direction transformation in Section 2.1 be denoted as

(13) $$\begin{array}{c} {\tilde{X}}^{\left(c\right)}={\left[{\tilde{x}}_{ij}^{\left(c\right)}\right]}_{m\times p},\; \end{array}$$

where m denotes the number of samples, p denotes the number of continuous features, and ${\tilde{x}}_{ij}^{\left(c\right)}$ denotes the normalized value of the j-th continuous feature in the i-th sample. The continuous operating feature vector of the i-th sample can be written as

(14) $$\begin{array}{c} {\tilde{x}}_{i}^{\left(c\right)}=\left[{\tilde{x}}_{i1}^{\left(c\right)},{\tilde{x}}_{i2}^{\left(c\right)},\ldots ,{\tilde{x}}_{ip}^{\left(c\right)}\right].\; \end{array}$$

Meanwhile, the continuous risk evidence matrix obtained after risk direction alignment in Section 2.1 is denoted as

(15) $$\begin{array}{c} R^{\left(c\right)}={\left[r_{ij}^{\left(c\right)}\right]}_{m\times p}.\; \end{array}$$

Here, $r_{ij}^{\left(c\right)}$ denotes the risk-oriented value of the j-th continuous feature in the i-th sample, and follows the unified convention that a larger value indicates higher potential risk. It should be noted that ${\tilde{X}}^{\left(c\right)}$ preserves the normalized operating structure of continuous variables, and is more suitable for learning the multivariable consistency relationship under normal photovoltaic operating conditions. In contrast, $R^{\left(c\right)}$ is mainly used to represent the risk direction and risk magnitude of continuous features. These two matrices play different roles in the subsequent risk screening process.

The basic assumption of continuous risk screening is that normal operating samples should be distributed near a relatively stable low dimensional operating manifold in the continuous feature space. In contrast, abnormal samples may deviate from this normal manifold due to shading, dust accumulation, hot spots, module degradation, or local electrical abnormalities. Therefore, potential risk at the continuous feature level can be quantified by learning the normal operating relationship and measuring the deviation degree of each sample.

#### 2.2.2. Construction of the Residual Autoencoding Model

To learn the normal operating consistency among continuous features, a residual autoencoding model is constructed. The model consists of an encoder and a decoder. The encoder compresses the normalized continuous operating features into a low dimensional hidden space, while the decoder reconstructs the input features from the hidden representation. The calculation process is expressed as

(16) $$\begin{array}{c} h_{i}=\phi \left(W_{e}{\tilde{x}}_{i}^{\left(c\right)}+b_{e}\right),\; \end{array}$$

(17) $$\begin{array}{c} {\hat{x}}_{i}^{\left(c\right)}=\psi \left(W_{d}h_{i}+b_{d}\right), \end{array}$$

where $h_{i}$ denotes the low dimensional hidden representation of the i-th sample, ${\hat{x}}_{i}^{\left(c\right)}$ denotes the reconstructed continuous operating feature vector, $W_{e}$ and $W_{d}$ denote the weight matrices of the encoder and decoder, respectively, $b_{e}$ and $b_{d}$ denote the bias terms, and ϕ(⋅) and ψ(⋅) denote nonlinear activation functions.

The autoencoding model is not used as a final classifier. Instead, it is used to learn the normal consistency structure among continuous variables. When a sample follows the normal photovoltaic operating pattern, its continuous features can be well reconstructed by the model. When low generation abnormality, abnormal module temperature rise, or abnormal conversion efficiency decrease occurs, the continuous feature combination deviates from the normal operating pattern, and the corresponding reconstruction error increases. Therefore, the reconstruction residual can be regarded as evidence of abnormal deviation at the continuous feature level.

To prevent abnormal samples from interfering with the learning of the normal operating manifold, the training samples of the autoencoding model are selected only from the training set. If reliable labels are available in the training set, normal samples are used for training. If labels are insufficient, an initial low risk sample set is constructed according to stable irradiance intervals, low power deviation, and normal temperature ranges within the training set. With this setting, the model is mainly forced to learn normal operating patterns rather than reconstruct abnormal patterns as normal states. Information leakage caused by the use of test set information is also avoided.

The training objective consists of reconstruction error, weight regularization, and sparse regularization of the hidden representation:

(18) $$\begin{array}{c} L_{AE}=\frac{1}{m_{0}}\sum_{i=1}^{m_{0}}{∥{\tilde{x}}_{i}^{\left(c\right)}-{\hat{x}}_{i}^{\left(c\right)}∥}_{2}^{2}+\lambda_{1}\left(∥W_{e}{∥}_{F}^{2}+∥W_{d}{∥}_{F}^{2}\right)+\lambda_{2}∥H{∥}_{1}, \end{array}$$

where $m_{0}$ denotes the number of normal or low risk samples used for training. The first term is the reconstruction error, which constrains the recovery capability of the model for continuous operating features. The second term is the weight regularization term, which is used to reduce overfitting. The third term is the sparse regularization term of the hidden representation, which encourages a more compact representation of operating states. $H=[h_{1},h_{2},\ldots ,h_{m_{0}}{]}^{T}$ denotes the hidden representation matrix of the training samples, and $\lambda_{1}$ and $\lambda_{2}$ are regularization coefficients.

**Figure 1:**
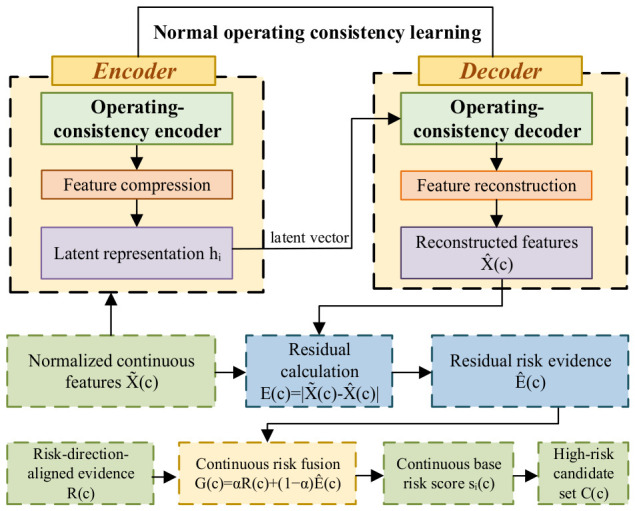
Schematic diagram of the operating-consistency residual autoencoding model.

The structure of the operating-consistency residual autoencoding model is illustrated in Figure 1.

#### 2.2.3. Quantification of Continuous Risk Residuals

After the model has been trained, the continuous operating features of all samples are input into the autoencoding model, and their reconstruction residuals are calculated. The residual of the j-th continuous feature in the i-th sample is defined as

(19) $$\begin{array}{c} e_{ij}^{\left(c\right)}=|{\tilde{x}}_{ij}^{\left(c\right)}-{\hat{x}}_{ij}^{\left(c\right)}|. \end{array}$$

Here, $e_{ij}^{\left(c\right)}$ represents the degree to which the corresponding continuous feature deviates from the normal operating consistency relationship. A larger residual indicates that the feature is more difficult to explain by the normal operating pattern, and thus has a higher abnormality degree.

Since $r_{ij}^{\left(c\right)}$ has already been transformed into the risk-oriented space, while $e_{ij}^{\left(c\right)}$ is the reconstruction residual obtained from the autoencoding model, their numerical scales are not completely consistent. Therefore, column wise normalization is first applied to the residual before fusion:

(20) $$\begin{array}{c} {\tilde{e}}_{ij}^{\left(c\right)}=\frac{e_{ij}^{\left(c\right)}-\underset{i}{min}\;e_{ij}^{\left(c\right)}}{\underset{i}{max}\;e_{ij}^{\left(c\right)}-\underset{i}{min}\;e_{ij}^{\left(c\right)}+\varepsilon }. \end{array}$$

Here, ${\tilde{e}}_{ij}^{\left(c\right)}\in [0,1]$ denotes the normalized continuous residual risk value. A larger value indicates a stronger deviation from the normal operating consistency relationship in the j-th continuous feature dimension.

If only the original risk oriented value is used, inconsistency among multiple variables may not be sufficiently captured. If only the reconstruction residual is used, the inherent risk magnitude of the feature itself may be ignored. Therefore, the continuous risk evidence after risk direction alignment and the normalized residual are jointly modeled, and the comprehensive continuous feature risk value is defined as

(21) $$\begin{array}{c} g_{ij}^{\left(c\right)}=\alpha r_{ij}^{\left(c\right)}+\left(1-\alpha \right){\tilde{e}}_{ij}^{\left(c\right)}, \end{array}$$

where α∈[0,1] is a balancing parameter. The first term $r_{ij}^{\left(c\right)}$ represents the risk magnitude of the original continuous feature after risk direction alignment, while the second term ${\tilde{e}}_{ij}^{\left(c\right)}$ represents the operating consistency deviation obtained from the autoencoding model. When α is larger, more emphasis is placed on the original risk intensity. When α is smaller, more emphasis is placed on the abnormal deviation from the normal operating manifold.

The parameter α is treated as a validation parameter. To prevent either the original risk magnitude or the residual deviation from completely dominating the continuous risk representation, the search range of α is limited to $\left[0.3,0.7\right]$. When α approaches 0.7, continuous risk representation places more emphasis on the original risk-oriented value. When α approaches 0.3, greater emphasis is placed on the operating consistency deviation reflected by the autoencoding residual. The final value is selected on the validation set according to the combined performance of AUC, Recall, and MCC, and its sensitivity is further analyzed in the experimental section.

To further ensure comparability among different continuous features, column wise normalization is applied to $g_{ij}^{\left(c\right)}$:

(22) $$\begin{array}{c} {\bar{g}}_{ij}^{\left(c\right)}=\frac{g_{ij}^{\left(c\right)}-\underset{i}{min}\;g_{ij}^{\left(c\right)}}{\underset{i}{max}\;g_{ij}^{\left(c\right)}-\underset{i}{min}\;g_{ij}^{\left(c\right)}+\varepsilon }. \end{array}$$

The continuous feature risk matrix is then obtained as

(23) $$\begin{array}{c} G^{\left(c\right)}={\left[{\bar{g}}_{ij}^{\left(c\right)}\right]}_{m\times p}. \end{array}$$

Each row of $G^{\left(c\right)}$ corresponds to one photovoltaic panel sample, and each column corresponds to one continuous feature dimension. Compared with $R^{\left(c\right)}$ in Section 2.1, $G^{\left(c\right)}$ not only retains risk direction information of continuous features, but also incorporates operating consistency residual information. Therefore, abnormal deviation at the continuous variable level can be more fully represented.

#### 2.2.4. Screening of Continuous High Risk Candidate Samples

To obtain sample level screening results from the continuous feature risk matrix, weighted aggregation is applied to the continuous features in $G^{\left(c\right)}$. Since different continuous features may have different discriminative capabilities for risk identification, an objective weighting method based on feature dispersion is adopted. For the j-th continuous feature, its standard deviation is defined as

(24) $$\begin{array}{c} \sigma_{j}^{\left(c\right)}=\sqrt{\frac{1}{m}\sum_{i=1}^{m}{\left({\bar{g}}_{ij}^{\left(c\right)}-{\bar{g}}_{\cdot j}^{\left(c\right)}\right)}^{2}}, \end{array}$$

where ${\bar{g}}_{\cdot j}^{\left(c\right)}$ denotes the mean value of the j-th continuous feature over all samples. A larger feature dispersion indicates stronger discriminative capability among samples, and thus a higher weight should be assigned. The continuous feature weight is defined as

(25) $$\begin{array}{c} \omega_{j}^{\left(c\right)}=\frac{\sigma_{j}^{\left(c\right)}}{\sum_{j=1}^{p}\sigma_{j}^{\left(c\right)}+\varepsilon }, \end{array}$$

with

(26) $$\begin{array}{c} \sum_{j=1}^{p}\omega_{j}^{\left(c\right)}=1. \end{array}$$

Based on the above weights, the continuous base risk score of the i-th sample is defined as

(27) $$\begin{array}{c} s_{i}^{\left(c\right)}=\sum_{j=1}^{p}\omega_{j}^{\left(c\right)}{\bar{g}}_{ij}^{\left(c\right)}. \end{array}$$

Here, $s_{i}^{\left(c\right)}$ represents the base risk level obtained from continuous features. This score reflects the abnormal degree of the photovoltaic panel in electrical output, environmental response, thermal state, and efficiency deviation. It should be emphasized that $\omega_{j}^{\left(c\right)}$ is used only for preliminary aggregation in the continuous risk screening stage, and does not represent the global indicator weight in the final composite risk ranking. The global risk weights are recalculated in the subsequent composite risk scoring stage. This setting avoids introducing excessive weighting assumptions at the screening stage, while leaving global weight learning to the final scoring stage.

An upper quantile threshold strategy is adopted for screening continuous high risk candidate samples. The basic idea is that samples with continuous base risk scores located in the upper tail of the score distribution are identified as candidate risk samples. Let the set of continuous base risk scores be defined as

(28) $$\begin{array}{c} S^{\left(c\right)}=\left\{s_{1}^{\left(c\right)},s_{2}^{\left(c\right)},\ldots ,s_{m}^{\left(c\right)}\right\}. \end{array}$$

The continuous risk screening threshold is then defined as

(29) $$\begin{array}{c} \tau_{c}=Q_{\eta }\left(S^{\left(c\right)}\right), \end{array}$$

where $Q_{\eta }(\cdot )$ denotes the quantile function, and η denotes the quantile level. To reduce the subjectivity caused by a fixed threshold, η is treated as a validation parameter and searched within $\left[0.85,0.95\right]$. This range corresponds to upper tail risk sample screening and provides a tradeoff between candidate sample coverage and false alarm rate. Unless otherwise specified, the initial quantile level is set to 0.90, meaning that samples with continuous base risk scores in the top 10 percent are preferentially screened as candidate risk samples.

When

(30) $$\begin{array}{c} s_{i}^{\left(c\right)}\geq \tau_{c}, \end{array}$$

the sample i is included in the preliminary high risk candidate set at the continuous feature level:

(31) $$\begin{array}{c} C^{\left(c\right)}=\left\{i|s_{i}^{\left(c\right)}\geq \tau_{c}\right\}. \end{array}$$

It should be noted that $C^{\left(c\right)}$ is not equivalent to the final high risk sample set. The purpose of continuous risk screening is to identify samples with abnormal deviation from the perspective of electrical and environmental operating variables, so that candidate samples can be provided for subsequent discrete state refinement. For example, some samples may show low power output, but if their corresponding irradiance is low or they are within a normal environmental fluctuation range, they may not represent true high risk states. In contrast, if such samples are also accompanied by shading state, hot spot indicators, equipment alarms, or abnormal maintenance records, their risk levels should be further increased in the subsequent stage.

In summary, the continuous risk screening process can be expressed as

$${\tilde{X}}^{\left(c\right)}\to {\hat{X}}^{\left(c\right)}\to E^{\left(c\right)}\to {\tilde{E}}^{\left(c\right)}\to G^{\left(c\right)}\to S^{\left(c\right)}\to C^{\left(c\right)}.$$

In this process, the normal operating consistency relationship among continuous features of photovoltaic panels is first learned. Then, abnormal deviation is characterized through reconstruction residuals, and a continuous feature risk matrix is formed by combining residual information with risk direction aligned continuous evidence. Finally, the continuous high risk candidate set $C^{\left(c\right)}$ and the discrete risk evidence matrix $R^{\left(d\right)}$ are jointly used as inputs to the subsequent discrete state refinement stage, so that a more reliable basis can be provided for composite risk ranking.

### 2.3. Candidate-Constrained Discrete-State Risk Enhancement Model

In the continuous risk screening stage, the continuous high-risk candidate set $C^{\left(c\right)}$ has been obtained based on continuous variables such as electrical output, environmental response, thermal state, and efficiency deviation. However, continuous features mainly reflect numerical deviations in the operating state of photovoltaic panels, and they may not sufficiently indicate whether such deviations are supported by state-level evidence.

For example, a sample may show reduced output power, but this phenomenon may be a normal fluctuation under low irradiance. If the same sample is also accompanied by a shading state, an abnormal module-temperature level, an increased power-deviation level, or equipment alarm information, its risk credibility and maintenance priority should be further increased. Therefore, after continuous risk screening, discrete state information is introduced to conduct state-level risk enhancement for continuous candidate samples.

A candidate-constrained discrete-state risk enhancement model is constructed in this study. The model does not perform independent discrete pattern mining over all samples. Instead, the continuous high-risk candidate set $C^{\left(c\right)}$ is taken as the main constrained scope, and whether different discrete states are enriched among continuous high-risk candidate samples is analyzed. If the occurrence proportion of a certain discrete state in the candidate set is clearly higher than its baseline occurrence proportion in all samples, this state may provide associative state evidence for continuous abnormality and strengthen risk assessment. In this way, discrete states are not treated as an independent parallel branch, but as state evidence supplement after continuous risk screening, which is consistent with the serial risk assessment framework proposed in this study.

**Figure 2:**
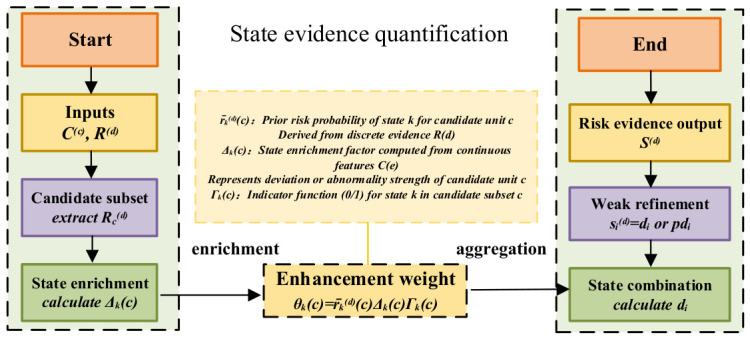
Flow chart of the candidate-constrained discrete-state risk enhancement model.

The overall process of the candidate-constrained discrete-state risk enhancement model is illustrated in Figure 2.

#### 2.3.1. Discrete-State Representation of Candidate Samples

Let the discrete risk evidence matrix obtained in Section 2.1 be denoted as

(32) $$\begin{array}{c} R^{\left(d\right)}={\left[r_{ik}^{\left(d\right)}\right]}_{m\times q}, \end{array}$$

where m denotes the number of samples, q denotes the number of discrete features, and $r_{ik}^{\left(d\right)}$ denotes the risk encoding value of the k-th discrete feature in the i-th sample. Discrete features may include state levels derived from continuous operating variables, equipment state flags, abnormal alarm records, inspection records, and maintenance records. When only continuous monitoring variables are available in practical data, discrete states can also be derived from irradiance level, module-temperature level, power-deviation level, efficiency-degradation level, and output-abnormality level. Such features are not directly represented as continuous numerical changes, but can provide structural information related to photovoltaic panel operating conditions, surface abnormalities, equipment states, and operation and maintenance events.

The continuous high-risk candidate set obtained in Section 2.2 is defined as

(33) $$\begin{array}{c} C^{\left(c\right)}=\left\{i|s_{i}^{\left(c\right)}\geq \tau_{c}\right\}. \end{array}$$

In the discrete-state risk enhancement stage, the candidate set $C^{\left(c\right)}$ is used as the main analysis scope. The discrete risk evidence submatrix corresponding to the candidate samples is expressed as

(34) $$\begin{array}{c} R_{C}^{\left(d\right)}={\left[r_{ik}^{\left(d\right)}\right]}_{i\in C^{\left(c\right)},\;k=1,\ldots ,q}. \end{array}$$

This matrix retains the discrete state information of continuous high-risk candidate samples and is used to analyze which states have stronger risk enhancement effects among continuous abnormal samples. It should be noted that $R^{\left(d\right)}$ in Section 2.1 is mainly used to describe the basic risk intensity of discrete states, while this section further focuses on the enrichment degree of discrete states in the continuous high-risk candidate set. These two parts are not repetitive. The former provides the risk meaning of the state itself, while the latter characterizes the incremental support of the state for continuous abnormal candidate samples.

#### 2.3.2. Candidate Enrichment Enhancement of Discrete States

To characterize the enhancement effect of discrete states on continuous candidate risk, the candidate-constrained state enrichment enhancement degree is defined. For a state c of the k-th discrete feature, the state-level basic risk value obtained by the risk encoding function in Section 2.1 is denoted as

$${\bar{r}}_{k}^{\left(d\right)}(c).$$

When the state of the k-th discrete feature in sample i is $c_{ik}$, its discrete risk evidence can be expressed as

(35) $$\begin{array}{c} r_{ik}^{\left(d\right)}={\bar{r}}_{k}^{\left(d\right)}\left(c_{ik}\right). \end{array}$$

Thus, $r_{ik}^{\left(d\right)}$ is the sample-level discrete risk evidence, whereas ${\bar{r}}_{k}^{\left(d\right)}(c)$ is the state-level basic risk encoding value. This definition allows the discrete risk encoding results in Section 2.1 to be directly used in the state enhancement calculation in this section.

For state c of the k-th discrete feature, its baseline occurrence proportion in all samples is first calculated as

(36) $$\begin{array}{c} P\left(x_{k}^{\left(d\right)}=c\right)=\frac{N_{k}(c)}{m}, \end{array}$$

where $N_{k}(c)$ denotes the number of samples in which the k-th discrete feature takes state c among all samples, and m denotes the total number of samples. This proportion reflects the baseline distribution of state c in the overall sample space.

Then, the candidate occurrence proportion of state c in the continuous high-risk candidate set $C^{\left(c\right)}$ is calculated as

(37) $$\begin{array}{c} P_{C}\left(x_{k}^{\left(d\right)}=c\right)=\frac{N_{C,k}(c)+\lambda P(x_{k}^{\left(d\right)}=c)}{|C^{\left(c\right)}|+\lambda }, \end{array}$$

where $N_{C,k}(c)$ denotes the number of samples in the candidate set whose k-th discrete feature takes state c, $|C^{\left(c\right)}|$ denotes the number of samples in the continuous high-risk candidate set, and λ is a smoothing coefficient. The term $\lambda P(x_{k}^{\left(d\right)}=c)$ is introduced to reduce the fluctuation of the candidate occurrence proportion under small-sample conditions and to make the candidate distribution estimate more stable.

Based on these two proportions, the candidate enrichment enhancement degree of state c is defined as

(38) $$\begin{array}{c} \Delta_{k}\left(c\right)=P_{C}\left(x_{k}^{\left(d\right)}=c\right)-P\left(x_{k}^{\left(d\right)}=c\right). \end{array}$$

When $\Delta_{k}(c)>0$, state c occurs more frequently in the continuous high-risk candidate set than in the overall sample space. In this case, the state can be regarded as having positive associative support for continuous abnormality. For example, if a hot-spot state, high-temperature level, or equipment alarm state has a low occurrence proportion in all samples but is clearly concentrated in the continuous high-risk candidate set, these states can provide additional state evidence for the continuous risk screening results. Conversely, when $\Delta_{k}(c)\leq 0$, the state does not show additional enrichment among continuous candidate samples and should not be regarded as a risk enhancement state.

To avoid unstable enhancement estimation caused by a small number of low-frequency states, a frequency reliability factor is further introduced:

(39) $$\begin{array}{c} \Gamma_{k}\left(c\right)=\frac{\log\left(1+N_{C,k}(c)\right)}{log\left(1+\underset{c}{max}\;N_{C,k}(c)\right)+\varepsilon }. \end{array}$$

Here, $\Gamma_{k}(c)\in [0,1]$. A larger value indicates that state c has higher statistical reliability in the candidate set. If no valid state record exists for a certain discrete feature in the candidate set, this feature is excluded from the risk enhancement calculation at this stage.

On this basis, the risk enhancement weight of state c is defined by combining the state-level basic risk encoding, candidate enrichment degree, and frequency reliability:

(40) $$\begin{array}{c} \theta_{k}\left(c\right)={\bar{r}}_{k}^{\left(d\right)}\left(c\right)\cdot \max\left(0,\Delta_{k}(c)\right)\cdot \Gamma_{k}\left(c\right). \end{array}$$

This equation considers three factors. First, ${\bar{r}}_{k}^{\left(d\right)}(c)$ represents the basic risk intensity of state c itself. Second, $max(0,\Delta_{k}(c))$ represents the positive enrichment degree of state c in the continuous high-risk candidate set. Third, $\Gamma_{k}(c)$ represents the statistical reliability of the occurrence frequency of this state. With this definition, only discrete states with basic risk meaning, candidate enrichment characteristics, and sufficient occurrence frequency are retained as effective risk enhancement states. Interference from irrelevant or accidental states is therefore reduced.

#### 2.3.3. State Combination Enhancement Under Candidate Constraints

A single discrete state may be insufficient to support photovoltaic panel risk judgment, while the co-occurrence of multiple states often reflects potential defects more effectively. For example, reduced output power alone may be caused by environmental fluctuation, and increased module temperature alone may also be related to external weather conditions. However, if a candidate sample is simultaneously associated with a high-temperature level, an increased power-deviation level, a hot-spot state, or equipment alarm records, the sample is more likely to have actual defect risk. Therefore, a state combination enhancement mechanism under candidate constraints is further constructed.

For sample i, the set of discrete states with risk enhancement effects is defined as

(41) $$\begin{array}{c} P_{i}=\left\{(k,c_{ik})|\theta_{k}(c_{ik})>0\right\}, \end{array}$$

where $c_{ik}$ denotes the specific state of sample i on the k-th discrete feature. The set $P_{i}$ represents all discrete states in this sample that can enhance continuous risk.

To integrate the joint effect of multiple discrete states, a complementary product form is used to define the sample-level discrete state enhancement intensity:

(42) $$\begin{array}{c} d_{i}=1-\prod_{(k,c_{ik})\in P_{i}}\left(1-\theta_{k}(c_{ik})\right). \end{array}$$

This equation is not used to assume strict mutual independence among different discrete states. Instead, it is used as a bounded nonlinear aggregation function. Its role is to increase the sample-level discrete enhancement intensity $d_{i}$ when multiple risk-enhancing states occur together. At the same time, the complementary product form restricts $d_{i}$ to the interval $\left[0,1\right]$, avoiding unbounded risk enhancement that may be caused by simple addition. If sample i does not contain any risk-enhancing state, namely $P_{i}$ is empty, then

(43) $$\begin{array}{c} d_{i}=0. \end{array}$$

In this case, the discrete states of this sample do not provide additional risk evidence, and its risk judgment should mainly rely on the continuous risk screening result and the subsequent composite scoring mechanism.

#### 2.3.4. Output of Discrete-State Risk Enhancement Evidence

After candidate-constrained discrete-state risk enhancement, each sample obtains a discrete state enhancement intensity $d_{i}$. Since a serial risk assessment framework is adopted in this study, continuous high-risk candidate samples should receive greater attention in the discrete-state refinement stage. At the same time, samples with insignificant continuous features but obvious discrete state abnormalities should not be completely ignored. Therefore, a weak refinement channel is introduced. The sample-level discrete-state risk enhancement score is defined as

(44) $$\begin{array}{c} s_{i}^{\left(d\right)}=\left\{\begin{array}{cc} d_{i}, & i\in C^{\left(c\right)},\\ \rho d_{i}, & i\notin C^{\left(c\right)}, \end{array}\right. \end{array}$$

where $\left.\rho \in [0,1\right)$ denotes the weak refinement coefficient for non-candidate samples. When a sample belongs to the continuous high-risk candidate set, its discrete state enhancement intensity is fully retained. When a sample does not belong to the continuous high-risk candidate set, its discrete state enhancement effect is appropriately reduced. The candidate constraint is reflected in two aspects. First, the state enhancement weight $\theta_{k}(c)$ is determined by the state enrichment degree in the continuous high-risk candidate set. Second, candidate samples retain the complete enhancement intensity, whereas non-candidate samples retain only the reduced auxiliary correction through ρ. This treatment maintains the serial logic of continuous screening followed by discrete refinement, while avoiding absolute neglect of non-candidate samples with strong discrete abnormal signals. The parameter ρ can be determined on the validation set. Unless otherwise specified, a small value is used to reflect the auxiliary correction role of discrete states for non-candidate samples.

Finally, the output of the discrete-state risk enhancement stage is expressed as

(45) $$\begin{array}{c} S^{\left(d\right)}=\left\{s_{1}^{\left(d\right)},s_{2}^{\left(d\right)},\ldots ,s_{m}^{\left(d\right)}\right\}. \end{array}$$

Here, $s_{i}^{\left(d\right)}$ denotes the risk enhancement evidence provided by discrete states for the i-th sample. It should be emphasized that $s_{i}^{\left(d\right)}$ is not the final composite risk score, but a state-level supplement to the continuous risk screening result. The continuous base risk score $s_{i}^{\left(c\right)}$ reflects the operating deviation of the sample in continuous variables such as electrical output, environmental response, thermal state, and efficiency deviation. The discrete-state risk enhancement score $s_{i}^{\left(d\right)}$ reflects whether this deviation is supported by discrete evidence, such as state levels, abnormal flags, alarm records, or operation and maintenance information. These two scores are fused in the subsequent composite risk scoring stage to obtain the final photovoltaic panel composite risk score and priority ranking result.

In summary, the candidate-constrained discrete-state risk enhancement process can be expressed as

$$\left(C^{\left(c\right)},R^{\left(d\right)}\right)\to \Delta_{k}(c)\to \theta_{k}(c)\to d_{i}\to S^{\left(d\right)}.$$

In this process, the continuous high-risk candidate set is first used as the constrained scope, and the enrichment effect of discrete states in candidate samples is calculated. Then, the basic risk encoding of discrete states and the frequency reliability factor are combined to suppress the influence of low-frequency accidental states. Finally, a bounded nonlinear state combination mechanism is used to form the sample-level discrete-state risk enhancement score. This score is used as an important input for composite risk scoring and ranking in Section 2.4.

### 2.4. CD-MABAC-Based Composite Risk Ranking Model

After continuous risk screening and discrete-state risk enhancement, two levels of risk evidence are obtained for each photovoltaic panel sample. The first is the continuous base risk score $s_{i}^{\left(c\right)}$, which is derived from continuous operating variables. The second is the discrete-state risk enhancement score $s_{i}^{\left(d\right)}$, which is obtained from discrete state information. Specifically, $s_{i}^{\left(c\right)}$ mainly reflects the abnormal degree of the sample in terms of electrical output, environmental response, thermal state, and efficiency deviation, whereas $s_{i}^{\left(d\right)}$ reflects whether such abnormality is supported by discrete evidence such as state levels, abnormal flags, alarm records, or operation and maintenance information.

However, a single risk score is insufficient to fully characterize the composite risk level of a sample. On the one hand, a sample with a high continuous risk score but weak discrete evidence may be affected by environmental fluctuation or short-term disturbance, and its risk credibility needs to be further examined. On the other hand, a sample with insignificant continuous deviation but strong discrete-state abnormality should not be completely ignored. Therefore, a Continuous-Discrete MABAC-based composite risk ranking model, referred to as CD-MABAC, is constructed in this study. In this model, continuous base risk, discrete-state risk enhancement, and continuous-discrete consistency risk are taken as three evaluation criteria. The Multi-Attributive Border Approximation Area Comparison method is then used to measure the deviation of each sample from the risk boundary, so that the final composite risk ranking can be obtained.

**Figure 3:**
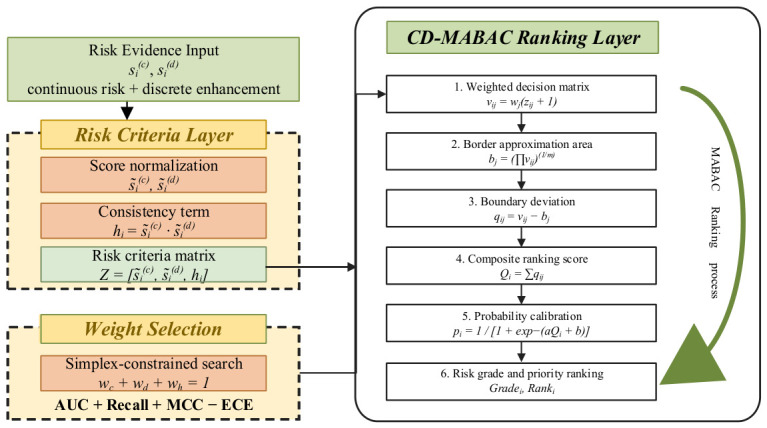
Flow chart of the CD-MABAC-based composite risk ranking model.

The overall process of the CD-MABAC-based composite risk ranking model is illustrated in Figure 3.

#### 2.4.1. Construction of the Continuous-Discrete Risk Criteria Matrix

Since $s_{i}^{\left(c\right)}$ and $s_{i}^{\left(d\right)}$ are obtained from different modeling stages, their numerical scales and distribution ranges may differ. To ensure fairness in the subsequent composite ranking process, the continuous base risk score and the discrete-state risk enhancement score are first normalized as

(46) $$\begin{array}{c} {\tilde{s}}_{i}^{\left(c\right)}=\frac{s_{i}^{\left(c\right)}-\underset{i}{min}\;s_{i}^{\left(c\right)}}{\underset{i}{max}\;s_{i}^{\left(c\right)}-\underset{i}{min}\;s_{i}^{\left(c\right)}+\varepsilon }, \end{array}$$

(47) $$\begin{array}{c} {\tilde{s}}_{i}^{\left(d\right)}=\frac{s_{i}^{\left(d\right)}-\underset{i}{min}\;s_{i}^{\left(d\right)}}{\underset{i}{max}\;s_{i}^{\left(d\right)}-\underset{i}{min}\;s_{i}^{\left(d\right)}+\varepsilon }. \end{array}$$

Here, ${\tilde{s}}_{i}^{\left(c\right)}$ denotes the normalized continuous base risk score, ${\tilde{s}}_{i}^{\left(d\right)}$ denotes the normalized discrete-state risk enhancement score, and ε is a small constant used to avoid division by zero.

To further characterize the consistency between continuous abnormality and discrete-state evidence, a continuous-discrete consistency term is introduced:

(48) $$\begin{array}{c} h_{i}={\tilde{s}}_{i}^{\left(c\right)}\cdot {\tilde{s}}_{i}^{\left(d\right)}. \end{array}$$

Here, $h_{i}$ denotes the continuous-discrete consistency risk of the i-th sample. It should be noted that $h_{i}$ is not introduced as a new independent data source. Instead, it is used as an interaction criterion to measure whether continuous risk and discrete-state evidence occur simultaneously. When both the continuous base risk and the discrete-state risk enhancement are high, $h_{i}$ increases accordingly, indicating that the sample not only shows abnormal deviation in continuous operating variables, but is also supported by discrete-state evidence. In this case, the risk credibility is higher. If the continuous risk is high but the discrete evidence is weak, or if the discrete-state abnormality is strong but the continuous deviation is not obvious, $h_{i}$ is not excessively amplified, so that risk misjudgment caused by single-source evidence can be reduced.

Based on the above three types of risk evidence, the continuous-discrete risk criteria matrix is constructed as

(49) $$\begin{array}{c} Z={\left[z_{ij}\right]}_{m\times 3}=\left[\begin{array}{ccc} {\tilde{s}}_{1}^{\left(c\right)} & {\tilde{s}}_{1}^{\left(d\right)} & h_{1}\\ {\tilde{s}}_{2}^{\left(c\right)} & {\tilde{s}}_{2}^{\left(d\right)} & h_{2}\\ \vdots & \vdots & \vdots \\ {\tilde{s}}_{m}^{\left(c\right)} & {\tilde{s}}_{m}^{\left(d\right)} & h_{m} \end{array}\right]. \end{array}$$

The first column represents the continuous base risk criterion, the second column represents the discrete-state risk enhancement criterion, and the third column represents the continuous-discrete consistency risk criterion. All three criteria are benefit-type risk criteria, meaning that a larger value indicates a higher risk level.

#### 2.4.2. Determination of Risk-Criteria Weights and Construction of the Weighted Decision Matrix

Different risk criteria do not contribute equally to the final composite risk ranking. The continuous base risk reflects the operating deviation of the sample, the discrete-state risk enhancement reflects the supporting strength of state evidence, and the continuous-discrete consistency risk reflects the synergistic enhancement between the two. Therefore, appropriate weights need to be assigned to the three risk criteria.

Let the risk-criteria weight vector be defined as

(50) $$\begin{array}{c} w=\left[w_{c},w_{d},w_{h}\right], \end{array}$$

subject to

(51) $$\begin{array}{c} w_{c}+w_{d}+w_{h}=1,w_{c},w_{d},w_{h}\geq 0. \end{array}$$

Here, $w_{c}$ denotes the weight of the continuous base risk criterion, $w_{d}$ denotes the weight of the discrete-state risk enhancement criterion, and $w_{h}$ denotes the weight of the continuous-discrete consistency risk criterion. To avoid bias caused by subjective weight assignment, w is treated as a validation parameter and searched under the simplex constraint:

(52) $$\begin{array}{c} w^{*}=\arg\underset{w}{max}J\left(w\right), \end{array}$$

(53) $$\begin{array}{c} s.t.w_{c}+w_{d}+w_{h}=1,w_{c},w_{d},w_{h}\geq 0. \end{array}$$

The objective function is defined as

(54) $$\begin{array}{c} J\left(w\right)=AUC+\beta_{1}Recall+\beta_{2}MCC-\beta_{3}ECE. \end{array}$$

This objective function jointly considers risk discrimination ability, abnormal sample recall, classification correlation, and probability calibration error. Specifically, AUC is used to reflect the overall discrimination ability, Recall emphasizes the detection ability for high-risk samples, MCC evaluates the overall classification performance under class imbalance, and ECE measures the probability calibration error. Unless otherwise specified, $\beta_{1}$, $\beta_{2}$, and $\beta_{3}$ are assigned equal weights to avoid artificial preference. The final weight combination is determined according to the comprehensive performance on the validation set.

After the weights have been determined, the MABAC weighted decision matrix is constructed as

(55) $$\begin{array}{c} {V=\left[v_{ij}\right]}_{m\times 3} \end{array}$$

where

(56) $$\begin{array}{c} v_{ij}=w_{j}\left(z_{ij}+1\right),j=1,2,3. \end{array}$$

Specifically, $w_{1}=w_{c}$, $w_{2}=w_{d}$, and $w_{3}=w_{h}$. Compared with directly using $w_{j}z_{ij}$, the form $z_{ij}+1$ avoids zero weighted risk values, which is beneficial for the subsequent geometric-mean calculation of the border approximation area. This form is also consistent with the commonly used construction of the weighted matrix in the MABAC method. The matrix V represents the weighted risk performance of each sample under different risk criteria and is used as the input for the subsequent calculation of the border approximation area.

#### 2.4.3. MABAC-Based Boundary Deviation Risk Ranking

The basic idea of the MABAC method is to construct a border approximation area and measure the deviation of each sample from the risk boundary. In the context of risk assessment, the border approximation area can be regarded as the reference risk level of the sample population under each risk criterion. If a sample is above this boundary level under several risk criteria, its overall risk is considered higher, and it should be assigned a higher maintenance priority. Conversely, if a sample is below the boundary level, its risk is considered relatively lower.

For the j-th risk criterion, the border approximation value is defined as

(57) $$\begin{array}{c} b_{j}={\left[\prod_{i=1}^{m}v_{ij}\right]}^{1/m}. \end{array}$$

Here, $b_{j}$ denotes the boundary risk level corresponding to the j-th risk criterion. The geometric mean is used to construct the border approximation value, which reduces the influence of extreme samples on the boundary level and allows the risk boundary to better reflect the overall distribution characteristics of the samples.

Then, the deviation of sample i from the border approximation area under the j-th risk criterion is calculated as

(58) $$\begin{array}{c} q_{ij}=v_{ij}-b_{j}. \end{array}$$

When $q_{ij}>0$, the weighted risk level of sample i under the j-th risk criterion is higher than the boundary risk level. When $q_{ij}<0$, the risk level of this sample under the corresponding criterion is lower than the boundary risk level. Thus, each sample can obtain its boundary-deviation performance under the three criteria of continuous base risk, discrete-state risk enhancement, and continuous-discrete consistency risk.

Finally, the CD-MABAC composite risk ranking score of sample i is defined as

(59) $$\begin{array}{c} Q_{i}=\sum_{j=1}^{3}q_{ij}.\; \end{array}$$

Here, $Q_{i}$ denotes the overall deviation degree of sample i from the continuous-discrete risk border approximation area. $Q_{i}$ can be either positive or negative. If $Q_{i}>0$, the sample is above the boundary risk region in the aggregate sense. If $Q_{i}<0$, the sample is below the boundary risk region in the aggregate sense. A larger $Q_{i}$ indicates that the sample is more strongly above the boundary level across multiple risk criteria, and therefore has a higher composite risk level. Accordingly, all samples are ranked in descending order of $Q_{i}$:

(60) $$\begin{array}{c} Rank=\mathrm{argsort}\left(-Q_{i}\right).\; \end{array}$$

The ranking result can be directly used to identify the risk priority of photovoltaic panels and to support subsequent inspection planning and maintenance resource allocation.

#### 2.4.4. Risk Probability Calibration and Risk Grade Classification

The MABAC ranking score $Q_{i}$ reflects the relative risk priority among samples, but it is not a probability value. To obtain an interpretable risk probability and support risk grade classification, a logistic calibration model is introduced to map the composite ranking score to a calibrated risk probability:

(61) $$\begin{array}{c} {\hat{p}}_{i}=\frac{1}{1+exp[-(aQ_{i}+b)]}. \end{array}$$

Here, ${\hat{p}}_{i}$ denotes the calibrated risk probability of the i-th sample, and a and b are calibration parameters. These parameters are fitted on the validation set using available fault labels, alarm labels, or constructed reference risk labels. Through logistic calibration, the original ranking score can be converted into a risk probability within $\left[0,1\right]$, which is more suitable for risk interpretation, graded warning, and evaluation of model calibration performance.

Based on the calibrated risk probability, the risk grade of each sample is further defined as

(62) $$\begin{array}{c} {\mathrm{Grade}}_{i}=\left\{\begin{array}{cc} \mathrm{Low}\;\mathrm{risk}, & {\hat{p}}_{i}<\tau_{1},\\ \mathrm{Medium}\;\mathrm{risk}, & \tau_{1}\leq {\hat{p}}_{i}<\tau_{2},\\ \mathrm{High}\;\mathrm{risk}, & {\hat{p}}_{i}\geq \tau_{2}. \end{array}\right. \end{array}$$

Here, $\tau_{1}$ and $\tau_{2}$ denote the grading thresholds between low, medium, and high risk. To reduce subjectivity in threshold setting, $\tau_{1}$ and $\tau_{2}$ are treated as validation parameters and determined by

(63) $$\begin{array}{c} \left(\tau_{1}^{*},\tau_{2}^{*}\right)=\arg\underset{\tau_{1},\tau_{2}}{max}J_{grade}\left(\tau_{1},\tau_{2}\right),0<\tau_{1}<\tau_{2}<1. \end{array}$$

The function $J_{grade}$ is used to evaluate the effectiveness of risk grade classification and can be constructed from Recall, MCC, false alarm rate, and risk distribution stability. With this setting, the risk grading thresholds are not directly specified by fixed empirical values. Instead, they are determined on the validation set by jointly considering high-risk sample detection, false alarm control, and rationality of the grade distribution. In practical operation and maintenance scenarios, the thresholds can also be adjusted according to maintenance resource constraints and risk tolerance.

Finally, the composite risk ranking result and the risk grade classification result jointly form the risk assessment output for photovoltaic panels. The ranking score $Q_{i}$ is mainly used to determine the relative maintenance priority among samples. The calibrated risk probability ${\hat{p}}_{i}$ provides an interpretable risk probability, while the risk grade ${\mathrm{Grade}}_{i}$ is used to form a graded warning result. In this way, both risk ranking and graded maintenance decision support can be achieved.

In summary, the CD-MABAC-based composite risk ranking process can be expressed as

$$\left(s_{i}^{\left(c\right)},s_{i}^{\left(d\right)}\right)\to \left({\tilde{s}}_{i}^{\left(c\right)},{\tilde{s}}_{i}^{\left(d\right)},h_{i}\right)\to Z\to V\to Q_{i}\to {\hat{p}}_{i}\to \left({\mathrm{Grade}}_{i},{\mathrm{Rank}}_{i}\right).$$

In this process, the continuous base risk and the discrete-state risk enhancement evidence are first transformed into a comparable scale. Then, the continuous-discrete consistency risk criterion is constructed. The MABAC method is subsequently used to calculate the overall deviation of each sample from the risk border approximation area and to obtain the final risk ranking score. Finally, risk probability and risk grade are obtained through logistic calibration and threshold-based classification. In this way, a complete risk assessment process is formed, covering continuous risk screening, discrete-state enhancement, composite risk ranking, and graded warning.

### 2.5. Methodological Workflow and Flowchart

To achieve systematic risk assessment and priority ranking of photovoltaic panels, this study proposes the OCERF framework, namely the Operating Consistency and Evidence Refinement Framework. The OCERF framework is formulated as a unified sequential risk-evidence transformation process. Each stage does not operate independently, but represents a different level of abstraction of the same risk-evidence modeling process: heterogeneous risk matrix construction, continuous risk screening, discrete-state evidence enhancement, CD-MABAC composite ranking, and risk grading.

First, continuous operating variables and discrete state variables are organized into a heterogeneous feature matrix. After missing data handling, normalization, risk-direction alignment, and discrete-state risk encoding, a unified risk-evidence matrix $R=[R^{\left(c\right)},R^{\left(d\right)}]$ is constructed.

Second, continuous risk screening is performed using the normalized continuous feature matrix ${\tilde{X}}^{\left(c\right)}$. An operating-consistency residual autoencoding model is used to learn normal relationships among continuous variables. Reconstruction residuals are combined with $R^{\left(c\right)}$ to obtain the continuous base risk score $s_{i}^{\left(c\right)}$, and an upper-quantile threshold is used to identify the continuous high-risk candidate set $C^{\left(c\right)}$.

Third, discrete-state evidence enhancement is conducted based on $R^{\left(d\right)}$ and $C^{\left(c\right)}$. Candidate-constrained enrichment analysis is used to identify discrete states concentrated in the continuous high-risk candidate set. By combining baseline risk encoding, candidate enrichment degree, and frequency reliability, the discrete-state enhancement score $s_{i}^{\left(d\right)}$ is obtained.

Fourth, the CD-MABAC model is used for composite risk ranking. The normalized continuous score, normalized discrete enhancement score, and consistency term $h_{i}={\tilde{s}}_{i}^{\left(c\right)}{\tilde{s}}_{i}^{\left(d\right)}$ are used to construct the risk criteria matrix. The MABAC boundary-deviation score $Q_{i}$ is then calculated to determine relative risk priority. $Q_{i}$ is mapped to a calibrated risk probability ${\hat{p}}_{i}$, and each sample is classified into low-, medium-, or high-risk levels.

**Figure 4:**
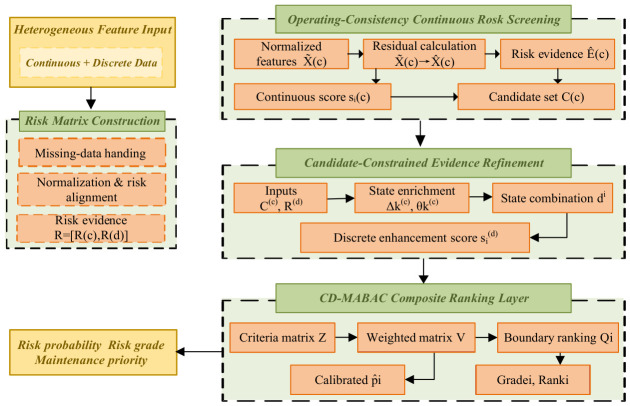
Overall workflow of the OCERF framework.

In summary, OCERF forms a complete process from heterogeneous feature construction to continuous screening, discrete evidence enhancement, composite ranking, and graded warning. The resulting outputs support photovoltaic panel inspection planning, maintenance priority allocation, and early risk intervention. The overall workflow is illustrated in Figure 4.

### 2.6. Parameter Definition and Sensitivity Analysis

The OCERF framework introduces four key parameters, α, η, ρ, and λ, which are used to regulate the weighting and fusion process of heterogeneous risk evidence. These parameters control the contribution strength of different evidence sources in the risk aggregation stage, rather than directly determining classification boundaries.

The parameter ranges are selected based on preliminary empirical analysis to ensure numerical stability and avoid extreme weighting conditions.

To evaluate robustness, sensitivity experiments are conducted by varying these parameters within reasonable ranges. The results demonstrate that the overall performance of the OCERF framework remains stable, indicating that the model is not overly sensitive to parameter tuning.

## 3. Empirical Case Study

### 3.1. Test Data and Risk-Label Construction

In this study, the Solar Power Generation Data dataset is used to evaluate the proposed OCERF framework for photovoltaic panel risk assessment. The dataset consists of generation-side records and weather-sensor observations collected from two photovoltaic plants. The generation data include timestamp, plant identifier, generation-unit identifier, DC and AC output power, and cumulative energy yield, while the weather data include ambient temperature, module temperature, and solar irradiation. The main variables used in this study are summarized in Table 2.

The dataset comprises operational and environmental measurements recorded at regular time intervals, forming a multivariate time-series dataset at the inverter level. Each record corresponds to a synchronized observation of electrical output and meteorological conditions, enabling consistent modeling of photovoltaic operating behavior under varying environmental conditions.

To ensure temporal consistency and reproducibility, generation and weather records are aligned using timestamp and plant identifier. Multiple inverter-level units within each plant are included, enabling fine-grained risk modeling and evaluation at the operational unit level.

Generation and weather records are matched according to DATE_TIME and PLANT_ID. The final dataset is organized into training, validation, and test sets using a chronological split strategy to avoid data leakage and ensure temporal consistency in model evaluation. Since weather measurements are provided at the plant level, they are assigned to all generation units within the same plant at each timestamp. Samples with near-zero irradiation are removed because low output power under non-generation conditions does not indicate photovoltaic risk.

The dataset used in this study includes two photovoltaic plants with different operational characteristics and environmental conditions, providing a multi-scenario evaluation setting for the proposed framework.

The continuous feature set $X^{\left(c\right)}$ is constructed from electrical output and environmental variables. Power deviation and conversion efficiency are further derived to characterize output mismatch and generation degradation. Since alarm records, hotspot annotations, soiling states, and maintenance labels are unavailable, the discrete state set $X^{\left(d\right)}$ is derived from continuous variables through engineering thresholds and quantile-based discretization. The resulting states include irradiation level, module-temperature level, power-deviation state, efficiency-degradation state, and output-abnormality state. These derived states are not used to replace continuous features, but to provide state-level evidence for the candidate-constrained refinement stage.

Because manually annotated fault labels are unavailable, reference risk labels are constructed according to photovoltaic operating rules. A sample is marked as reference high risk when abnormal low output occurs under effective irradiation, when high module temperature is accompanied by efficiency degradation, when power deviation falls into the upper abnormal quantile of the training set, or when low output, high irradiation, and high module temperature occur simultaneously. These labels are used for training-set risk encoding, validation-based parameter selection, probability calibration, and comparative evaluation, but they are not treated as the direct output of OCERF.

To prevent information leakage, training, validation, and test sets are divided before parameter estimation. Normalization parameters, discretization thresholds, and reference-label construction thresholds are determined from the training set, while hyperparameters and calibration parameters are selected using the validation set. All parameters are then fixed for final test evaluation. The final outputs of OCERF include $Q_{i}$, ${\hat{p}}_{i}$, $Grade_{i}$, and $Rank_{i}$.

### 3.2. Experimental Environment and Parameter Settings

All experiments were conducted on a Windows 11 operating system with an Intel Core i5 processor and 16 GB of memory. Data preprocessing, feature construction, model calculation, performance evaluation, and result visualization were mainly implemented in MATLAB R2024a. A fixed random seed was used during data splitting, parameter search, and model calculation to improve reproducibility.

After invalid low-irradiation samples were removed, the photovoltaic operating samples were divided into training, validation, and test sets with a ratio of 70%, 15%, and 15%, respectively. The training set was used for normalization, discretization, residual autoencoder training, and state-level risk encoding. The validation set was used for hyperparameter selection, CD-MABAC weight determination, and probability calibration. The test set was used only for final performance evaluation.

For the continuous risk screening module, a fully connected residual autoencoder was constructed using the normalized continuous feature matrix ${\tilde{X}}^{\left(c\right)}$. Reconstruction residuals were calculated to measure deviations from normal operating consistency and were fused with continuous risk evidence to obtain the continuous base risk score. The fusion coefficient α was selected from $\left[0.3,0.7\right]$ according to validation performance.

For the candidate-constrained discrete-state refinement module, the candidate threshold η was selected from $\left\{0.85,0.90,0.95\right\}$, and the weak refinement coefficient ρ was selected from $\left[0.1,0.3\right]$. The smoothing coefficient used in state-level risk encoding and candidate enrichment estimation was also determined on the validation set to reduce the influence of low-frequency states.

For the CD-MABAC composite ranking module, the risk criteria consisted of the normalized continuous risk score, the normalized discrete enhancement score, and the continuous-discrete consistency term. The criteria weight vector was denoted as $w=[w_{c},w_{d},w_{h}]$, with $w_{c}+w_{d}+w_{h}=1$. A simplex-constrained grid search with a step size of 0.05 was used to select the optimal weight combination according to validation performance. Logistic calibration and risk-grade thresholds were then determined on the validation set and fixed for test evaluation.

The key parameter settings of the proposed OCERF framework and the comparison models are summarized in Table 3.

The above experimental setting was adopted to ensure that OCERF and all comparison models were evaluated under the same data partition and validation protocol. All hyperparameters were determined using only the training and validation sets, while the test set remained unseen during model development. Therefore, the final test results can reflect the generalization performance of different models under unseen photovoltaic operating samples.

### 3.3. Validation Method

To verify the effectiveness of the proposed OCERF framework, several representative baseline models were compared under identical data partition and parameter selection protocols. The selected baseline models include Logistic Regression, SVM, Random Forest, and BP Neural Network. All models were trained on the same training set, tuned on the same validation set, and finally evaluated on the same test set. The reference risk labels constructed in Section 3.1 were used only for model validation and performance comparison. For all models, the output risk scores were converted into comparable risk probabilities or ranking scores before evaluation.

The performance of the models was comprehensively evaluated from three perspectives: discrimination ability, probability calibration quality, and inspection priority ranking ability. Specifically, ROC curve and AUC were used to evaluate the overall separability between reference high risk samples and normal samples. PR curve and PR-AUC were adopted to further evaluate the identification ability for the high-risk class under imbalanced data distribution. The confusion matrix was used to show threshold-based classification results. Brier Score and ECE were adopted to assess the reliability of predicted risk probabilities. Precision@k, Hit@k, and Lift@k were further introduced to evaluate whether high risk photovoltaic samples could be concentrated in the top ranked inspection region. The evaluation metrics are described as follows.

1. ROC Curve and AUC Metric

The receiver operating characteristic curve was used to evaluate the discrimination ability of different models under varying decision thresholds. The ROC curve is drawn by taking the false positive rate as the horizontal axis and the true positive rate as the vertical axis. When the curve is closer to the upper left corner, reference high risk samples and normal samples are considered to be better separated. Based on the ROC curve, the area under the curve is calculated as a threshold independent performance indicator. A larger AUC value indicates stronger overall discrimination ability and better robustness to threshold variation.

2. PR Curve and PR-AUC Metric

The precision recall curve was adopted to further evaluate the identification performance for the high-risk class. In photovoltaic risk assessment, reference high risk samples usually account for a smaller proportion of the dataset than normal samples. Under such imbalanced conditions, the PR curve can provide a more direct assessment of whether high risk samples are effectively detected. The PR curve is drawn by taking Recall as the horizontal axis and Precision as the vertical axis. PR-AUC is calculated as the area under the PR curve. A larger PR-AUC value indicates that the model can maintain a higher precision while capturing more high risk samples, which is important for reducing missed risks and unnecessary inspections.

3. Confusion Matrix

The confusion matrix was adopted to provide an intuitive description of classification results under a selected decision threshold. It contains four basic components: true positives, false positives, true negatives, and false negatives. In the photovoltaic risk assessment task, true positives denote reference high-risk samples correctly identified by the model, while false negatives denote high-risk samples that are missed. False positives indicate normal or low-risk samples incorrectly classified as high risk, which may cause unnecessary inspection.

The confusion matrix is defined as

(64) $$\begin{array}{c} C=\left[\begin{array}{cc} TP & FN\\ FP & TN \end{array}\right], \end{array}$$

where TP, FN, FP, and TN represent true positives, false negatives, false positives, and true negatives, respectively. Based on the confusion matrix, several classification indicators can be calculated as

(65) $$\begin{array}{c} Accuracy=\frac{TP+TN}{TP+TN+FP+FN}, \end{array}$$

(66) $$\begin{array}{c} Recall=\frac{TP}{TP+FN},\; \end{array}$$

(67) $$\begin{array}{c} Precision=\frac{TP}{TP+FP}, \end{array}$$

(68) $$\begin{array}{c} F_{1}=\frac{2\cdot \mathrm{Precision}\cdot \mathrm{Recall}}{\mathrm{Precision}+\mathrm{Recall}}. \end{array}$$

Therefore, the confusion matrix can directly reflect the tradeoff between missed risk detection and redundant maintenance actions. In this study, the confusion matrix is mainly used to evaluate whether more reference high-risk photovoltaic samples can be captured under the same inspection budget.

4. Brier Score

The Brier Score was used to evaluate the accuracy of predicted risk probabilities. Unlike ranking metrics that only focus on relative order, the Brier Score measures the numerical difference between the predicted probability and the reference risk label. It is defined as

(69) $$\begin{array}{c} Brier=\frac{1}{N}\sum_{i=1}^{N}({\hat{p}}_{i}-y_{i}{)}^{2}, \end{array}$$

where N denotes the number of test samples, ${\hat{p}}_{i}$ denotes the calibrated risk probability of the i-th sample, and $y_{i}\in \{0,1\}$ denotes the reference risk label. A smaller Brier Score indicates that the predicted probabilities are closer to the reference labels and that the probabilistic risk estimation is more accurate.

5. Expected Calibration Error

The expected calibration error was introduced to measure the consistency between predicted risk probabilities and observed high risk frequencies. The test samples were divided into M probability bins according to their predicted probabilities. For each bin, the average predicted confidence and the observed high-risk frequency were calculated. ECE is defined as

(70) $$\begin{array}{c} ECE=\sum_{m=1}^{M}\frac{|B_{m}|}{N}|acc\left(B_{m}\right)-conf\left(B_{m}\right)|, \end{array}$$

where $B_{m}$ denotes the sample set in the m-th probability bin, $|B_{m}|$ denotes the number of samples in that bin, $\mathrm{acc}(B_{m})$ denotes the observed proportion of reference high risk samples, and $\mathrm{conf}(B_{m})$ denotes the average predicted probability. A smaller ECE value indicates better probability calibration. For photovoltaic maintenance, well calibrated probabilities are important because they allow operators to interpret model outputs as risk levels rather than only as relative scores.

6. Precision@k

Precision@k was used to evaluate the purity of the top ranked high-risk region. Let $T_{k}$ denote the set of top k samples ranked by the predicted risk score, and let I(⋅) denote the indicator function. Precision@k is defined as

(71) $$\begin{array}{c} Precision@k=\frac{\sum_{i\in T_{k}}I(y_{i}=1)}{k}. \end{array}$$

This metric measures the proportion of reference high risk samples among the top k predicted samples. A higher Precision@k means that the highest ranked samples contain fewer normal samples and are more suitable for prioritized inspection.

7. Hit@k

Hit@k was adopted to measure the proportion of all reference high risk samples captured within the top k ranked samples. Let $N_{+}$ denote the total number of reference high risk samples in the test set. Hit@k is defined as

(72) $$\begin{array}{c} Hit@k=\frac{\sum_{i\in T_{k}}I(y_{i}=1)}{N_{+}}. \end{array}$$

This metric reflects the high-risk capture ability under limited inspection resources. In practical photovoltaic operation and maintenance, only a limited number of panels, inverters, or operating samples can be checked first. Therefore, a higher Hit@k indicates that more risk related samples can be captured when only the top ranked samples are inspected.

8. Lift@k

Lift@k was used to quantify the improvement of model guided inspection compared with random inspection. It is defined as

(73) $$\begin{array}{c} Lift@k=\frac{\mathrm{Precision}@k}{N_{+}/N}, \end{array}$$

where $N_{+}/N$ represents the overall reference high risk ratio in the test set. A Lift@k value greater than 1 indicates that the model performs better than random selection. A larger Lift@k value means that the model can concentrate high risk photovoltaic samples more effectively in the top ranked region, which is especially useful for maintenance priority allocation.

In summary, ROC and AUC were used to measure overall discrimination ability, PR curve and PR-AUC were used to evaluate high risk class identification under imbalanced data distribution, the confusion matrix was used to analyze classification results under a selected threshold or a fixed inspection-budget setting, Brier Score and ECE were used to evaluate the reliability of predicted risk probabilities, and Precision@k, Hit@k, and Lift@k were used to assess inspection priority ranking ability. This validation setting is consistent with the objective of photovoltaic risk assessment, where both accurate high-risk identification and reliable maintenance prioritization are required.

### 3.4. Comprehensive Evaluation Results

**Figure 5:**
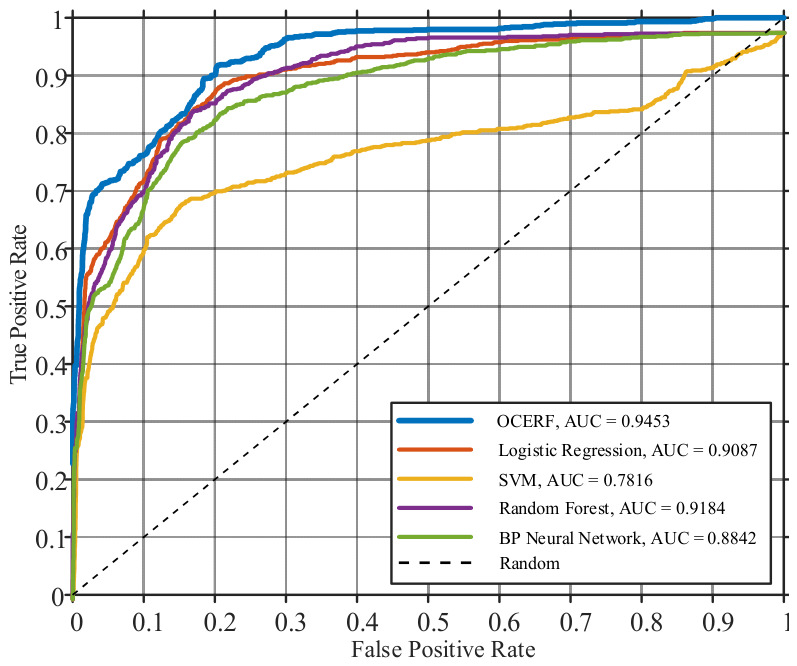
ROC curves of OCERF and comparison models.

As shown in Figure 5, the ROC curves of OCERF and the four comparison models were used to evaluate the discrimination ability for reference high-risk photovoltaic samples. The ROC curve of OCERF is generally located above those of the comparison models, especially in the low false-positive-rate region. This indicates that more potential high-risk samples can be identified while the false alarm level is kept relatively low. The corresponding AUC values are listed in Table 4. OCERF achieves the highest AUC value of 0.9436, while the AUC values of Random Forest, Logistic Regression, BP Neural Network, and SVM are 0.9187, 0.9079, 0.8842, and 0.7816, respectively.

These results indicate that the combination of operating-consistency continuous risk screening, candidate-constrained discrete-state evidence refinement, and CD-MABAC-based composite ranking improves the separability between high-risk and normal photovoltaic samples. Compared with the best baseline model, Random Forest, the AUC of OCERF is increased by 0.0249. Compared with Logistic Regression, BP Neural Network, and SVM, the AUC improvements are 0.0357, 0.0594, and 0.1620, respectively. Therefore, the effectiveness of the proposed framework in identifying high-risk photovoltaic samples is verified.

**Figure 6:**
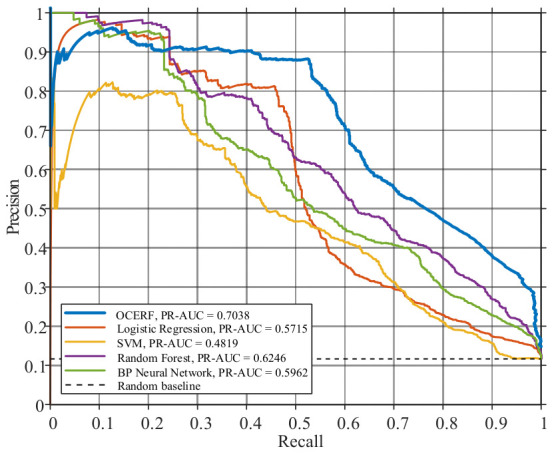
PR curves of OCERF and comparison models.

In addition to the ROC analysis, the PR curves were further used to evaluate the identification performance for reference high-risk photovoltaic samples under imbalanced data distribution. As shown in Figure 6, the PR curve of OCERF is generally located above those of the comparison models over a wide recall range. This indicates that a higher precision can be maintained when more high-risk samples are identified. The corresponding PR-AUC values are listed in Table 5. OCERF obtains the highest PR-AUC value of 0.7038, while the PR-AUC values of Random Forest, Logistic Regression, BP Neural Network, and SVM are 0.6246, 0.5962, 0.5715, and 0.4819, respectively.

Compared with the ROC curve, the PR curve provides a more direct evaluation of high-risk sample identification, because the proportion of reference high-risk samples is relatively limited in the photovoltaic risk assessment task. The superior PR-AUC of OCERF indicates that high-risk samples are more effectively ranked in the front part of the risk list, while the proportion of incorrectly selected normal samples is reduced. This result is consistent with the ROC analysis and further confirms that the proposed framework has stronger discrimination and ranking ability for photovoltaic risk assessment.

Overall, the PR-AUC results further demonstrate that OCERF is more suitable for maintenance-oriented risk screening, where high-risk photovoltaic samples are expected to be ranked before normal samples under limited inspection resources.

**Figure 7:**
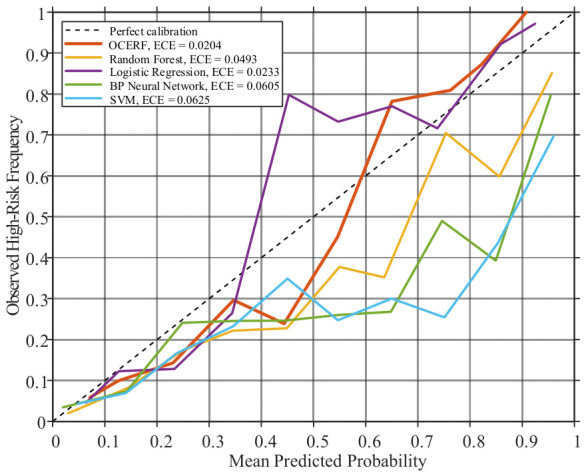
Calibration curves of OCERF and comparison models.

As shown in Figure 7, calibration curves were used to evaluate whether the predicted risk probabilities were consistent with the observed high-risk frequencies. The diagonal dashed line represents perfect calibration, and a curve closer to this line indicates better probability reliability. The corresponding Brier Score and ECE values are summarized in Table 6.

OCERF achieves the lowest Brier Score of 0.06819 and the lowest ECE of 0.02037 among all compared models. Logistic Regression also shows competitive calibration performance, with a Brier Score of 0.06864 and an ECE of 0.02328, while Random Forest, BP Neural Network, and SVM present larger calibration errors. These results indicate that the calibrated probabilities of OCERF are more consistent with the actual high-risk frequency. Combined with the ROC and PR results, OCERF not only improves discrimination and ranking performance, but also provides more reliable probabilistic outputs for photovoltaic risk assessment.

To further evaluate the classification results under limited maintenance resources, confusion matrices were constructed under the same inspection budget. For each model, the same number of samples with the highest predicted risk scores was classified as high risk, so that the comparison focused on high-risk capture ability under a fixed inspection capacity.

**Figure 8:**
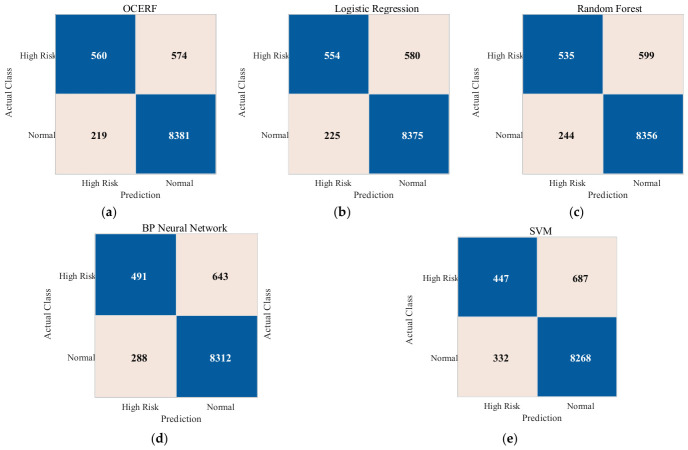
Confusion matrices of OCERF and comparison models under the same inspection budget: (**a**) OCERF; (**b**) Logistic Regression; (**c**) Random Forest; (**d**) BP Neural Network; (**e**) SVM.

As shown in Figure 8, OCERF correctly identifies 560 high-risk samples, which is higher than Logistic Regression, Random Forest, BP Neural Network, and SVM. Meanwhile, fewer normal samples are incorrectly selected by OCERF than by most comparison models. These results are consistent with the ROC and PR analyses, and further indicate that OCERF is more suitable for maintenance-priority allocation under limited inspection resources.

**Figure 9:**
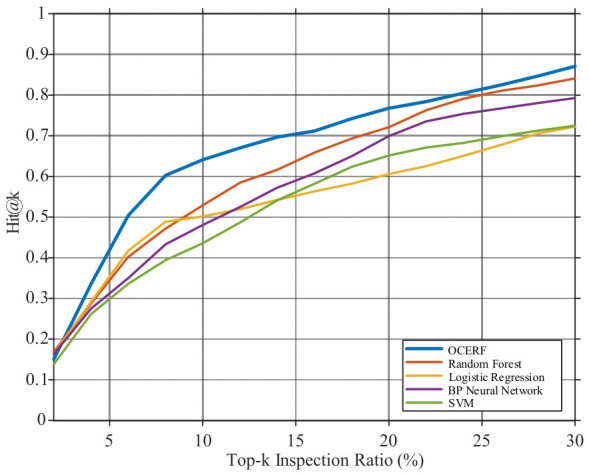
Hit@k curves of OCERF and comparison models. Different colors represent different models.

To further evaluate the maintenance-priority ranking ability of different models, Hit@k curves were plotted under different top-k inspection ratios. As shown in Figure 9, Hit@k increases gradually as the inspection ratio becomes larger, because more samples are included in the prioritized inspection set. Compared with the baseline models, OCERF maintains a higher Hit@k value over most inspection ratios, indicating that more reference high-risk photovoltaic samples can be captured when the same proportion of samples is selected for priority inspection.

The corresponding Precision@k and Lift@k results are summarized in Table 7 and Table 8, respectively. At the Top 10% inspection ratio, OCERF obtains a Precision@k of 0.7456 and a Lift@k of 6.4000, both of which are higher than those of the comparison models. At the Top 20% and Top 30% inspection ratios, OCERF still maintains competitive and generally superior ranking performance. These results indicate that OCERF can concentrate high-risk photovoltaic samples more effectively in the front part of the risk ranking list. This is consistent with the PR curve and confusion matrix results, and further confirms that the proposed framework is suitable for maintenance-priority allocation under limited inspection resources.

To improve the interpretability of the reported evaluation results, the proposed OCERF framework provides a structured decomposition of risk evidence into continuous and discrete components. The continuous risk component captures operating-consistency deviations derived from electrical and environmental measurements, while the discrete component represents state-level abnormal indicators reflecting operational conditions.

In addition, the enhancement weight θ_k_(c) enables the quantification of the contribution of each discrete state for a given candidate unit. By jointly analyzing π_k_^(d)(c), Δ_k_(c), and I_k_(c), the model provides a traceable explanation of whether high-risk predictions are primarily driven by continuous operational deviations or reinforced by discrete abnormal states.

This decomposition establishes an explicit interpretability path from raw sensor measurements to final risk ranking results, improving transparency and practical usability of the OCERF framework in photovoltaic maintenance applications.

### 3.5. Statistical Significance Analysis

To evaluate the robustness and statistical stability of the proposed OCERF framework, a bootstrap resampling strategy is applied on the test set. In each iteration, samples are drawn with replacement, and AUC values are computed for OCERF and all baseline models. This process is repeated for 1000 bootstrap iterations to approximate the empirical distribution of model performance under data variability.

Based on the bootstrap samples, the mean AUC, standard deviation, and 95% confidence interval (CI) are reported for all methods. The statistical results are consistent with the bootstrap-based ranking and provide supplementary evidence supporting the reliability of the observed performance differences.

To further support the reliability of performance differences, pairwise DeLong tests are conducted between OCERF and each baseline model using predicted scores. The statistical results are consistent with the bootstrap-based ranking and provide supplementary evidence that the observed performance improvements are not due to random sampling fluctuations.

**Figure 10:**
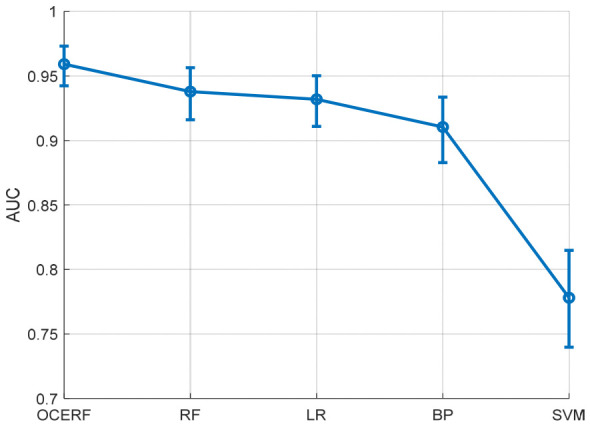
Distribution of AUC values with 95% confidence intervals obtained from bootstrap resampling on the test set.

The bootstrap-based AUC distributions and corresponding 95% confidence intervals are shown in Figure 10.

### 3.6. Label Construction Robustness Analysis

To further evaluate the reliability of the proposed framework, we investigate the robustness of the rule-based risk label construction strategy under different threshold configurations. Since the proposed OCERF framework relies on operational proxy labels rather than ground-truth fault annotations, it is necessary to verify whether the labeling process is sensitive to parameter selection.

In this study, a sensitivity analysis is conducted by varying key quantile thresholds used in irradiance filtering and power deviation definition. Three different configurations are tested to represent different levels of labeling strictness.

**Figure 11:**
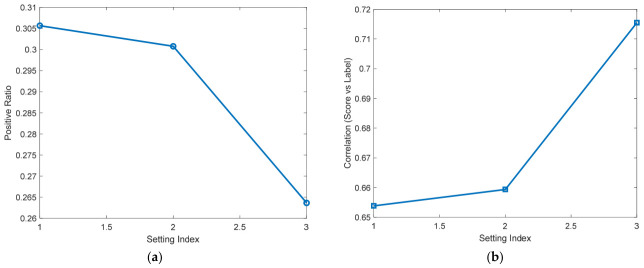
Performance Analysis of the Proposed OCERF Framework under Different Evaluation Metrics. (**a**) Discrimination and ranking performance; (**b**) Probability calibration performance.

The results are illustrated in Figure 11, where Figure 11a shows the variation of positive sample ratio under different settings, and Figure 11b presents the correlation between constructed labels and model-derived risk scores.

As observed, although stricter thresholds lead to a reduction in the proportion of positive samples, the overall variation remains limited and follows a consistent trend. More importantly, the correlation between constructed labels and risk scores remains stable across all configurations, indicating that the constructed labels are not overly sensitive to threshold selection.

These results demonstrate that the proposed labeling strategy is robust and provides consistent risk patterns for subsequent model training and evaluation. This further supports the reliability of the proxy-label-based learning framework adopted in this study.

To evaluate the robustness of parameter selection, a sensitivity analysis is conducted by varying α, η, ρ, and λ within a reasonable range. The results indicate that the overall performance of the OCERF framework remains stable under different parameter configurations, demonstrating that the model is not overly sensitive to parameter tuning.

### 3.7. Ablation Study

To verify the contribution of each component in OCERF, an ablation study was conducted by removing or replacing key modules. The complete framework was compared with four variants: OCERF without operating-consistency screening, OCERF without discrete-state evidence refinement, OCERF without CD-MABAC ranking, and OCERF without probability calibration. All variants were evaluated under the same data partition and validation protocol.

**Figure 12:**
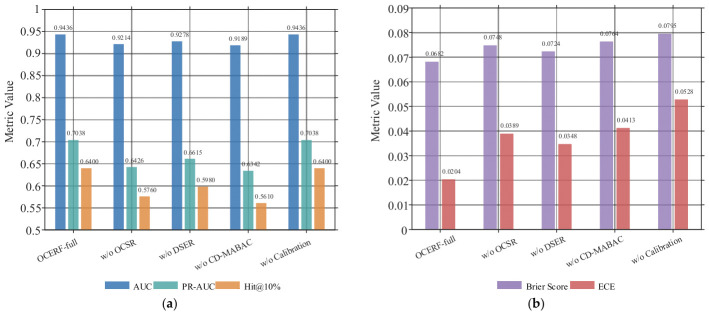
Ablation study results of the proposed OCERF framework: (**a**) Discrimination and ranking metrics; (**b**) Probability calibration metrics.

As shown in Figure 12, OCERF-full achieves the best overall performance, with an AUC of 0.9436, a PR-AUC of 0.7038, a Brier Score of 0.06819, an ECE of 0.02037, and a Hit@10% of 0.6400. Removing operating-consistency screening or discrete-state evidence refinement leads to clear declines in AUC, PR-AUC, and Hit@10%, indicating that both continuous residual evidence and discrete-state enhancement contribute to high-risk sample identification. When CD-MABAC is replaced, the ranking-related metrics decrease further, showing the effectiveness of the boundary-deviation ranking mechanism. In addition, removing probability calibration does not change AUC, PR-AUC, or Hit@10%, but increases Brier Score and ECE, which confirms its role in improving probability reliability. Overall, the ablation results demonstrate that each module contributes to OCERF, and the complete framework provides the most balanced performance.

### 3.8. Sensitivity Analysis

To evaluate the robustness of OCERF to key hyperparameters, a sensitivity analysis was conducted on three parameters, including the continuous evidence fusion coefficient α, the candidate screening quantile η, and the weak refinement coefficient ρ. AUC and PR-AUC were used as evaluation metrics, so that both overall discrimination ability and high-risk identification performance could be assessed.

**Figure 13:**
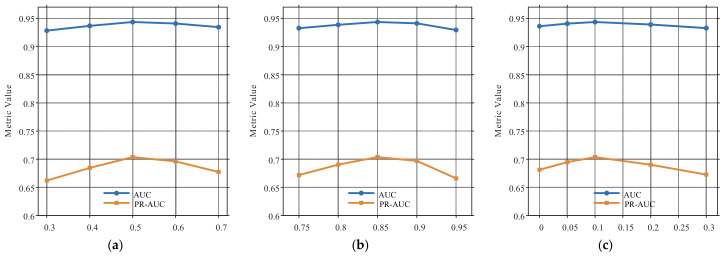
Sensitivity analysis of key parameters in OCERF: (**a**) sensitivity to α; (**b**) sensitivity to η; (**c**) sensitivity to ρ.

As shown in Figure 13, OCERF maintains relatively stable performance within the tested parameter ranges. For α, the best performance is obtained around 0.50. When α is too small, the model relies excessively on reconstruction residual evidence, while the original risk-oriented continuous evidence is weakened. When α is too large, the contribution of operating-consistency residuals becomes insufficient. For η, the performance first increases and then decreases, with the best result obtained around 0.85. A low η introduces more low-risk samples into the candidate set, while an overly high η may exclude some potential high-risk samples. For ρ, moderate values provide better performance, because a small amount of discrete-state evidence retained for non-candidate samples can improve ranking stability, whereas excessive retention weakens the candidate-constrained enhancement mechanism.

Overall, the sensitivity results indicate that OCERF does not depend on a single extreme parameter setting. The framework achieves stable performance over a reasonable parameter range, which confirms its robustness for photovoltaic risk assessment.

### 3.9. Cross-Plant Evaluation

To evaluate the generalization capability of the proposed OCERF framework under heterogeneous operating conditions, a cross-plant evaluation is conducted using data from two photovoltaic plants with different environmental and operational characteristics.

**Figure 14:**
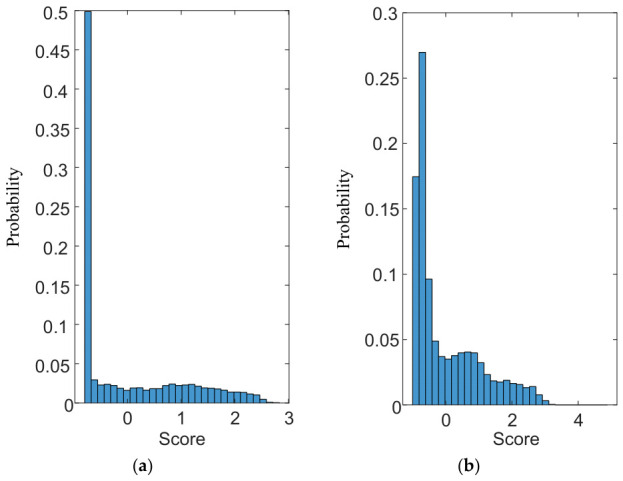
Distribution comparison of risk scores across two photovoltaic plants: (**a**) Plant 1 Risk Score; (**b**) Plant 2 Risk Score.

As shown in Figure 14, the risk score distributions of the two plants exhibit noticeable differences. Plant 1 presents a more concentrated distribution, whereas Plant 2 shows a more dispersed pattern with a heavier tail. This indicates the presence of a clear domain shift between different operating environments, which is a common challenge in real-world photovoltaic monitoring systems.

To quantitatively assess cross-plant consistency, the Spearman rank correlation coefficient between the risk scores of the two plants is computed, resulting in a value of −0.3753. This moderate negative correlation suggests that risk rankings derived from a single plant are not directly transferable to another plant without adaptation.

The observed discrepancy further highlights the importance of consistency-aware modeling in OCERF, where operating-condition alignment and evidence refinement are designed to mitigate distributional differences across sites.

Overall, the results demonstrate that significant cross-site heterogeneity exists in photovoltaic operating data, and the proposed framework is capable of addressing this challenge through improved risk representation stability.

### 3.10. Computational Complexity and Deployment Analysis

To evaluate the practical feasibility of the proposed OCERF framework, a computational complexity and deployment analysis is conducted.

The overall computational complexity of OCERF is approximately linear with respect to the number of samples, as each module operates independently on sample-level risk evidence transformation and does not require pairwise comparisons. The operating-consistency screening and discrete-state refinement modules introduce only lightweight feature-level operations, while the ranking stage based on CD-MABAC involves simple matrix aggregation and boundary deviation calculation.

In terms of deployment, the framework is suitable for cloud-edge collaborative photovoltaic monitoring systems. The continuous feature screening and discrete-state enhancement modules can be executed at the edge layer for real-time preprocessing, while the final risk ranking and calibration are performed in the cloud layer to ensure computational efficiency and model consistency.

Overall, the proposed OCERF framework maintains low computational overhead and is compatible with real-world photovoltaic monitoring scenarios.

### 3.11. Spatial Case Study and Interpretability Analysis

A spatial case study is conducted to interpret OCERF risk outputs in real photovoltaic deployment environments. Sample-level composite risk scores are aggregated and mapped onto deployment regions, enabling spatial-level interpretation without introducing additional modeling components.

**Figure 15:**
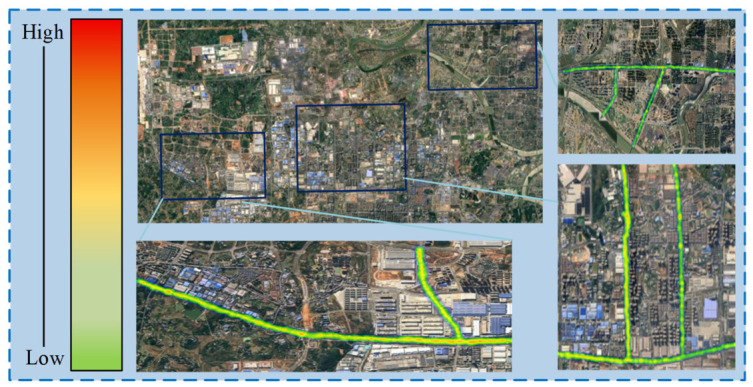
Spatial visualization of OCERF-based photovoltaic risk distribution across deployment regions.

As shown in Figure 15, the results exhibit clear spatial heterogeneity. High-risk regions are clustered along specific operational corridors and industrial zones, while low-risk regions are mainly located in stable operating areas. Moderate-risk regions appear as transitional zones, reflecting gradual degradation patterns.

Zoom-in analysis shows that high-risk regions are associated with persistent power deviations, abnormal temperature rise, and unstable irradiance response, which are consistent with degradation behaviors such as partial shading and thermal accumulation. In contrast, low-risk regions maintain stable electrical–environmental consistency.

These spatial patterns are consistent with the risk representations learned by OCERF from heterogeneous indicators. Overall, the spatial projection demonstrates that OCERF outputs are both spatially structured and physically interpretable, supporting practical maintenance prioritization.

To further enhance the engineering interpretability of high-risk predictions, the identified spatial risk patterns can be associated with typical photovoltaic degradation mechanisms, including partial shading, thermal accumulation, and equipment aging. These mechanisms are consistent with observed indicators such as persistent power deviations, abnormal temperature increases, and unstable irradiance responses.

## 4. Limitations and Future Research Directions

However, these labels are not intended to represent true fault conditions, but rather simplified operational risk proxies for comparative analysis. 

The proposed OCERF framework improves photovoltaic risk assessment by integrating heterogeneous risk evidence, operating-consistency residual modeling, candidate-constrained discrete-state refinement, and CD-MABAC-based composite ranking. The experimental results show that the framework can provide effective high-risk identification, probability calibration, and maintenance-priority ranking. However, several aspects still require further improvement.

First, because manually annotated fault records are unavailable in the public dataset, reference risk labels are constructed according to photovoltaic operating rules. These labels can capture deviations in operating behavior such as low output under similar environmental conditions, high-temperature low-efficiency operation, and excessive power deviation, but they may not fully cover complex defect mechanisms in real photovoltaic plants. Second, the present study mainly relies on generation data and weather-sensor data. Some important factors, such as dust accumulation, shading, hotspot evolution, string mismatch, inverter alarms, and maintenance behavior, are still represented indirectly through derived variables or discretized states. Finally, the CD-MABAC weights and risk-grade thresholds are mainly determined through validation-based search, and their adaptability under different plants, seasons, and operating conditions still requires further investigation.

Future research can be conducted in the following directions:

- More reliable risk-label construction can be developed by combining rule-based labels with expert inspection records and verified fault events, so that the evaluation results can better reflect real photovoltaic degradation processes.
- Multimodal photovoltaic condition-monitoring data can be further incorporated, including image, thermal, electrical, weather, and maintenance-text information, to improve the completeness of risk evidence and enhance the interpretability of discrete-state refinement.
- Online learning and cloud-edge collaborative updating strategies can be explored to support continuous model calibration, dynamic risk-grade adjustment, and real-time maintenance-priority recommendation in large-scale photovoltaic plants.

Overall, this study focuses on relative risk ranking rather than fault-level diagnosis, which should be considered when interpreting the results.

Further validation on datasets from different climatic regions and module technologies will be an important direction for future work to enhance generalization capability.

## 5. Conclusions

For accurate identification, calibrated reference-risk assessment, and maintenance-priority ranking of latent risks in photovoltaic panels, an Operating Consistency and Evidence Refinement Framework (OCERF) is proposed for photovoltaic risk assessment and maintenance prioritization. The framework integrates photovoltaic generation data and weather sensor data, and is designed to produce a composite risk score, calibrated risk probability, and risk ranking. The main contributions are summarized as follows:

Heterogeneous risk representation: Continuous operating variables and derived discrete states are transformed into a unified risk-evidence matrix through missing-data handling, normalization, risk-direction alignment, and discrete-state encoding, providing a consistent input for subsequent risk screening and ranking.

Operating-consistency risk screening: A residual autoencoding model is used to characterize relationships among electrical output, environmental response, thermal state, and efficiency-related variables. Reconstruction residuals are combined with continuous risk evidence to indicate deviations from normal operating patterns.

Evidence refinement and composite ranking: A candidate-constrained discrete-state refinement mechanism is introduced to enhance state-level evidence representation for high-risk candidates. CD-MABAC further integrates continuous risk signals, discrete state information, and their consistency to derive reference risk probabilities, risk grades, and relative priority rankings.

Systematic evaluation and engineering applicability: ROC, PR-AUC, Brier Score, ECE, confusion matrix, and Top-k ranking metrics are used for comprehensive evaluation. Ablation and sensitivity analyses are conducted to assess module contributions and parameter robustness. The results indicate that OCERF can improve the consistency of high-risk sample ranking, probability estimation quality, and maintenance-priority ordering compared with baseline methods.

## Figures and Tables

**Table 1:** Representative heterogeneous features for photovoltaic-panel risk assessment.

Feature Category	Subcategory	Representative Variables	Data Type
Electrical operating variables	Output state	DC voltage, DC current, output power, power deviation, conversion efficiency	Continuous
Environmental variables	External condition	Solar irradiance, ambient temperature, module temperature, humidity, wind speed	Continuous
Surface condition variables	Panel surface state	Dust level, shading condition, soiling state, hotspot indicator	Discrete
Equipment status variables	Operation state	Inverter status, string state, bypass diode status, protection-action flag	Discrete
Fault and maintenance variables	Event information	Fault type, alarm type, inspection record, cleaning/maintenance flag	Discrete

**Table 2:** Main fields of the Solar Power Generation Data dataset used in this study.

Data Source	Field	Description	Use in This Study
Generation data	DATE_TIME	Sampling timestamp	Data matching
Generation data	PLANT_ID	Photovoltaic plant identifier	Data matching
Generation data	SOURCE_KEY	Generation unit or inverter identifier	Unit-level sample construction
Generation data	DC_POWER	DC-side output power	Continuous feature
Generation data	AC_POWER	AC-side output power	Continuous feature
Generation data	DAILY_YIELD	Daily accumulated generation	Auxiliary continuous feature
Generation data	TOTAL_YIELD	Total accumulated generation	Auxiliary cumulative feature
Weather data	AMBIENT_TEMPERATURE	Ambient temperature	Continuous feature
Weather data	MODULE_TEMPERATURE	Module temperature	Continuous feature
Weather data	IRRADIATION	Solar irradiation	Continuous feature and sample filtering

**Table 3:** Key parameter settings of the OCERF framework and comparison models.

Model or Module	Parameter	Value or Search Range
Experimental platform	Main software	MATLAB R2024a
Data partition	Training, validation, and test sets	70%, 15%, and 15%
Randomness control	Random seed	Fixed random seed
Residual autoencoder	Network structure	Fully connected encoder and decoder
Residual autoencoder	Maximum epochs	100
Residual autoencoder	Early stopping patience	10
Residual autoencoder	Hidden dimension	Selected using the validation set
Continuous risk fusion	α	$\left[0.3,0.7\right]$
Candidate screening	η	$\left\{0.85,0.90,0.95\right\}$
Discrete state refinement	ρ	$\left[0.1,0.3\right]$
CD-MABAC	Weight search step	0.05
CD-MABAC	Criteria weights	$w_{c}+w_{d}+w_{h}=1$
Probability calibration	Calibration method	Logistic calibration
Comparison model	Logistic Regression	Logistic classification model
Comparison model	SVM	RBF kernel
Comparison model	Random Forest	Decision tree ensemble model
Comparison model	BP Neural Network	Fully connected neural network

**Table 4:** AUC values of OCERF and comparison models.

Model	AUC
OCERF	0.9436
Random Forest	0.9187
Logistic Regression	0.9079
BP Neural Network	0.8842
SVM	0.7816

**Table 5:** PR-AUC values of OCERF and comparison models.

Model	PR-AUC
OCERF	0.7038
Random Forest	0.6246
Logistic Regression	0.5962
BP Neural Network	0.5715
SVM	0.4819

**Table 6:** Calibration performance of OCERF and comparison models.

Model	Brier Score	ECE
OCERF	0.06819	0.02037
Logistic Regression	0.06864	0.02328
Random Forest	0.07179	0.04933
BP Neural Network	0.08388	0.06046
SVM	0.09342	0.06253

**Table 7:** Precision@k values of OCERF and comparison models.

Model	Top 5%	Top 10%	Top 20%	Top 30%
OCERF	0.9786	0.7456	0.4485	0.3378
Random Forest	0.7922	0.6174	0.4194	0.3262
Logistic Regression	0.8155	0.5825	0.3553	0.2796
BP Neural Network	0.7223	0.5592	0.4077	0.3068
SVM	0.6990	0.5126	0.3786	0.2796

**Table 8:** Lift@k values of OCERF and comparison models.

Model	Top 5%	Top 10%	Top 20%	Top 30%
OCERF	8.4000	6.4000	3.8500	2.9000
Random Forest	6.8000	5.3000	3.6000	2.8000
Logistic Regression	7.0000	5.0000	3.0500	2.4000
BP Neural Network	6.2000	4.8000	3.5000	2.6333
SVM	6.0000	4.4000	3.2500	2.4000

## Data Availability

The data used in this study were obtained from the publicly available Solar Power Generation Data dataset. The dataset is available online at: https://www.kaggle.com/datasets/anikannal/solar-power-generation-data (accessed on 6 June 2026).

## References

[1] Abdulla H., Sleptchenko A., Nayfeh A. (2024). Photovoltaic systems operation and maintenance: A review and future directions. Renew. Sustain. Energy Rev..

[2] Keisang K., Bader T., Samikannu R. (2021). Review of operation and maintenance methodologies for solar photovoltaic microgrids. Front. Energy Res..

[3] Hijjawi U., Lakshminarayana S., Xu T., Fierro G.P.M., Rahman M. (2023). A review of automated solar photovoltaic defect detection systems: Approaches, challenges, and future orientations. Sol. Energy.

[4] El-Banby G.M., Moawad N.M., Abouzalm B.A., Abouzaid W.F., Ramadan E.A. (2023). Photovoltaic system fault detection techniques: A review. Neural Comput. Appl..

[5] Mansouri M., Trabelsi M., Nounou H., Nounou M. (2021). Deep learning-based fault diagnosis of photovoltaic systems: A comprehensive review and enhancement prospects. IEEE Access.

[6] Li B., Delpha C., Diallo D., Migan-Dubois A. (2021). Application of artificial neural networks to photovoltaic fault detection and diagnosis: A review. Renew. Sustain. Energy Rev..

[7] Mellit A., Kalogirou S.A. (2021). Artificial intelligence and internet of things to improve efficacy of diagnosis and remote sensing of solar photovoltaic systems: Challenges, recommendations and future directions. Renew. Sustain. Energy Rev..

[8] Høiaas I., Grujic K., Imenes A.G., Burud I., Olsen E., Belbachir N. (2022). Inspection and condition monitoring of large-scale photovoltaic power plants: A review of imaging technologies. Renew. Sustain. Energy Rev..

[9] Manno D., Cipriani G., Ciulla G., Di Dio V., Guarino S., Lo Brano V. (2021). Deep learning strategies for automatic fault diagnosis in photovoltaic systems by thermographic images. Energy Convers. Manag..

[10] Yuan Z., Xiong G., Fu X. (2022). Artificial Neural Network for Fault Diagnosis of Solar Photovoltaic System: A Survey. Energies.

[11] Madeti S.R., Singh S.N. (2017). A comprehensive study on different types of faults and detection techniques for solar photovoltaic system. Sol. Energy.

[12] Chine W., Mellit A., Lughi V., Malek A., Sulligoi G., Pavan A.M. (2016). A novel fault diagnosis technique for photovoltaic systems based on artificial neural networks. Renew. Energy.

[13] Garoudja E., Harrou F., Sun Y., Kara K., Chouder A., Silvestre S. (2017). Statistical fault detection in photovoltaic systems. Sol. Energy.

[14] Silvestre S., Chouder A., Karatepe E. (2013). Automatic fault detection in grid connected PV systems. Sol. Energy.

[15] Chouder A., Silvestre S. (2010). Automatic supervision and fault detection of PV systems based on power losses analysis. Energy Convers. Manag..

[16] Zhao Y., Yang L., Lehman B., de Palma J.F., Mosesian J., Lyons R. Decision tree-based fault detection and classification in solar photovoltaic arrays. Proceedings of the 2012 Twenty-Seventh Annual IEEE Applied Power Electronics Conference and Exposition (APEC).

[17] Firth S.K., Lomas K.J., Rees S.J. (2010). A simple model of PV system performance and its use in fault detection. Sol. Energy.

[18] Cortes C., Vapnik V. (1995). Support-vector networks. Mach. Learn..

[19] Breiman L. (2001). Random forests. Mach. Learn..

[20] Hinton G.E., Salakhutdinov R.R. (2006). Reducing the dimensionality of data with neural networks. Science.

[21] LeCun Y., Bengio Y., Hinton G. (2015). Deep learning. Nature.

[22] Hochreiter S., Schmidhuber J. (1997). Long short-term memory. Neural Comput..

[23] Chen T., Guestrin C. XGBoost: A scalable tree boosting system. Proceedings of the 22nd ACM SIGKDD International Conference on Knowledge Discovery and Data Mining.

[24] He K., Zhang X., Ren S., Sun J. Deep residual learning for image recognition. Proceedings of the IEEE Conference on Computer Vision and Pattern Recognition.

[25] Kingma D.P., Ba J. (2014). Adam: A method for stochastic optimization. arXiv.

[26] Deitsch S., Christlein V., Berger S., Buerhop-Lutz C., Maier A., Gallwitz F., Riess C. (2019). Automatic classification of defective photovoltaic module cells in electroluminescence images. Sol. Energy.

[27] Tsanakas J.A., Ha L., Al Shakarchi F. (2017). Advanced inspection of photovoltaic installations by aerial triangulation and terrestrial georeferencing of thermal/visual imagery. Renew. Energy.

[28] Akram M.W., Li G., Jin Y., Chen X., Zhu C., Zhao X., Khaliq A., Faheem M., Ahmad A. (2019). CNN based automatic detection of photovoltaic cell defects in electroluminescence images. Energy.

[29] Harrou F., Sun Y., Taghezouit B., Saidi A., Hamlati M.E. (2018). Reliable fault detection and diagnosis of photovoltaic systems based on statistical monitoring approaches. Renew. Energy.

[30] Saaty T.L. (2008). Decision making with the analytic hierarchy process. Int. J. Serv. Sci..

[31] Chen C.T. (2000). Extensions of the TOPSIS for group decision-making under fuzzy environment. Fuzzy Sets Syst..

[32] Opricovic S., Tzeng G.H. (2004). Compromise solution by MCDM methods: A comparative analysis of VIKOR and TOPSIS. Eur. J. Oper. Res..

[33] Pamučar D., Ćirović G. (2015). The selection of transport and handling resources in logistics centers using Multi-Attributive Border Approximation Area Comparison (MABAC). Expert Syst. Appl..

[34] Diakoulaki D., Mavrotas G., Papayannakis L. (1995). Determining objective weights in multiple criteria problems: The CRITIC method. Comput. Oper. Res..

[35] Mardani A., Jusoh A., Zavadskas E.K., Cavallaro F., Khalifah Z. (2015). Sustainable and renewable energy: An overview of the application of multiple criteria decision making techniques and approaches. Sustainability.

[36] Kabak M., Dağdeviren M. (2014). Prioritization of renewable energy sources for Turkey by using a hybrid MCDM methodology. Energy Convers. Manag..

[37] Büyüközkan G., Güleryüz S. (2017). Evaluation of renewable energy resources in Turkey using an integrated MCDM approach with linguistic interval fuzzy preference relations. Energy.

[38] Yazdani M., Zarate P., Zavadskas E.K., Turskis Z. (2019). A combined compromise solution method for group decision-making problems. Expert Syst. Appl..

[39] Vaswani A., Shazeer N., Parmar N., Uszkoreit J., Jones L., Gomez A.N., Kaiser Ł., Polosukhin I. (2017). Attention is all you need. Advances in Neural Information Processing Systems (NeurIPS).

[40] Zhou H., Zhang S., Peng J., Zhang S., Li J., Xiong H., Zhang W. (2021). Informer: Beyond efficient transformer for long sequence time-series forecasting. Proc. AAAI Conf. Artif. Intell..

[41] Wu H., Xu J., Wang J., Long M. (2021). Autoformer: Decomposition transformers with auto-correlation for long-term series forecasting. Adv. Neural Inf. Process. Syst..

[42] Lundberg S.M., Lee S.I. (2017). A unified approach to interpreting model predictions. Advances in Neural Information Processing Systems.

[43] Lundberg S.M., Erion G.G., Chen H., DeGrave A., Prutkin J.M., Nair B., Katz R., Himmelfarb J., Bansal N., Lee S.-I. (2019). From local explanations to global understanding with explainable AI for trees. Nat. Mach. Intell..

[44] Arrieta A.B., Díaz-Rodríguez N., Del Ser J., Bennetot A., Tabik S., Barbado A., Garcia S., Gil-Lopez S., Molina D., Benjamins R. (2020). Explainable Artificial Intelligence (XAI): Concepts, taxonomies, opportunities and challenges toward responsible AI. Inf. Fusion.

[45] Eldele E., Ragab M., Chen Z., Wu M., Kwoh C.K., Li X., Guan C. Time-Series Representation Learning via Temporal and Contextual Contrasting. Proceedings of the Thirtieth International Joint Conference on Artificial Intelligence.

[46] Nie Y., Nguyen N.H., Sinthong P., Kalagnanam J. A time series is worth 64 words: Long-term forecasting with transformers (PatchTST). Proceedings of the International Conference on Learning Representations (ICLR).

[47] Brier G.W. (1950). Verification of forecasts expressed in terms of probability. Mon. Weather Rev..

[48] Niculescu-Mizil A., Caruana R. Predicting good probabilities with supervised learning. Proceedings of the 22nd International Conference on Machine Learning.

[49] Naeini M.P., Cooper G., Hauskrecht M. Obtaining well calibrated probabilities using Bayesian binning. Proceedings of the Twenty-Ninth AAAI Conference on Artificial Intelligence.

[50] Guo C., Pleiss G., Sun Y., Weinberger K.Q. (2017). On calibration of modern neural networks. arXiv.

[51] Kull M., Silva Filho T.M., Flach P. (2017). Beyond sigmoids: How to obtain well-calibrated probabilities from binary classifiers with beta calibration. Electron. J. Stat..

